# A Comprehensive Cohort Description and Statistical Grouping of Community-Based Residential Rehabilitation Service Users in Australia

**DOI:** 10.3389/fpsyt.2019.00798

**Published:** 2019-11-08

**Authors:** Stephen Parker, Dan Siskind, Daniel F. Hermens, Frances Dark, Gemma McKeon, Nicole Korman, Urska Arnautovska, Meredith Harris, Harvey Whiteford

**Affiliations:** ^1^Rehabilitation Academic Clinical Unit, Metro South Addiction and Mental Health Services (MSAMHS), Brisbane, QLD, Australia; ^2^School of Public Health, University of Queensland, Herston, QLD, Australia; ^3^Sunshine Coast Mind and Neuroscience–Thompson Institute, University of the Sunshine Coast, Birtinya, QLD, Australia; ^4^Psychosis Academic Clinical Unit, Metro South Addiction and Mental Health Services (MSAMHS), Brisbane, QLD, Australia; ^5^PA Foundation, Princess Alexandra Hospital, Brisbane, QLD, Australia

**Keywords:** community care unit, rehabilitation, residential care, schizophrenia, severe and persistent mental illness

## Abstract

**Background:** Community Care Units (CCUs) are a model of community-based residential rehabilitation support available in Australia that assists people affected by severe and persistent mental illness to enhance their independent living skills and community involvement. These services have been subject to limited evaluation, and available descriptions of consumer cohorts lack relevance to the understanding of their rehabilitation needs.

**Method:** A clinical assessment battery covering a broad range of relevant domains was completed with consumers commencing at three CCUs in Queensland, Australia, between December 2014 and December 2017 (N = 145). The cohort was described based on demographic, diagnostic, treatment-related variables, and the assessment battery. The comparability of included sites was assessed. This contemporary cohort was also compared to the pooled cohort of Australian community-based residential rehabilitation services emerging from a previous systematic review. Additionally, cluster analysis (CA) was completed in two stages based on the clinician-rated assessments: hierarchical CA (Wards method) to identify the optimal number of clusters, followed by K-means clustering.

**Results:** Dominant features of the cohort were male sex and the primary diagnoses of schizophrenia spectrum disorders. The average consumer age was 31.4 years. Most consumers were referred from the community, had been living with family, and were not subject to involuntary treatment orders. No site-based differences were observed on demographic, diagnostic and treatment-related variables. However, some site-based variation in levels of symptoms and functional impairment emerged. Overall, the cohort was comparable with the Transitional Residential Rehabilitation (TRR) cohort defined in a previous systematic review. Through CA, a three-cluster solution emerged: Cluster 1 (15%) was characterised by higher levels of substance use comorbidity; Cluster 2 (39%) was characterised by higher levels of disability and symptoms; and Cluster 3 (46%) was distinguished by lower levels of general psychiatric symptoms.

**Conclusions:** The cohort was generally comparable to the TRR cohort. Site-based variability in the characteristics of admitted consumers was minimal. The CA solution suggested that three different sub-groups of consumers are admitted to CCUs, which have implications for adapting the approach to rehabilitation. Recommendations include ensuring early availability of interventions to address co-morbidities and pacing rehabilitation expectations to consumers stage of recovery.

## Introduction

Community Rehabilitation Units are public mental health services that provide time-limited recovery-oriented clinical rehabilitation support in a community residential setting (1). Most people who access mental health rehabilitation services in Australia and the United Kingdom are diagnosed with schizophrenia or a related psychotic disorder. Interventions provided by mental health rehabilitation services are complex and focus on improving psychosocial functioning while optimising clinical recovery (2). Care planning is done collaboratively and is personalised to the individual consumer’s goals. Treatment is provided over an extended period, with an expectation of iterative progress towards multiple and changing goals. Service models, such as the Community Care Unit (CCU) model in Queensland and Victoria, have become increasingly available in Australia over the past 20 years despite limited research being available about patterns in service utilisation and the outcomes achieved for consumers (1). Planning the nature of care delivered at these services could be improved through clarification of the clinical and functional profiles of admitted consumers.

A recent systematic review found that descriptions of service users of Community Rehabilitation Units in Australia are generally limited to demographic and diagnostic information (1). This review defined contemporary services (operating from the early 2000s) as the Transitional Residential Rehabilitation (TRR) type. TRR service consumers were predominantly males, aged in their mid-30s, with a primary diagnosis of schizophrenia or a related psychotic disorder. Approximately half of these consumers were subject to an involuntary treatment order and the majority were referred from a community mental health service. These consumers demonstrate high levels of global impairment and psychosocial disability both at the commencement of rehabilitation and throughout the period of care. Although relevant, this information is insufficient to direct an understanding of what evidence-based rehabilitation interventions should be prioritised. Comprehensive rehabilitation assessment includes consideration of each consumer’s symptoms, cognition, functional capacity, stage of recovery, social environment, strengths, coping strategies, and personal goals (3, 4).

Our qualitative analysis of staff perceptions of the work of an Australian Community Rehabilitation Unit suggested that they make intuitive distinctions between consumers who are and are not “rehabilitation ready” (5). The concept of readiness was linked to ideas about who should and should not be admitted to the CCU. Characteristics staff associated with not being ready included symptomatic acuity, active substance use, and accommodation instability. Additionally, staff identified deficits in their skills to support consumers with issues relating to youth mental health, substance use, and acute symptoms affecting consumers transferred from inpatient care. Clarifying the profile of people who utilise CCUs could inform policy and planning decisions regarding the adequacy of current models of care, and their role in the mental health services array.

The present study aims to provide a comprehensive description of service users at three CCUs in Queensland, Australia. Two of these units were trialling a novel “integrated staffing model” where peer support workers rather than nursing staff occupy the majority of staff roles (6). Additionally, cluster analysis (CA) was conducted with a view towards identifying whether any meaningful consumer subgroups could be characterised in line with the intuitive groupings qualitatively described by staff. CA is a statistically driven approach to classification within multivariate data sets that generates clusters (groups of cases) by maximizing the similarity of cases within each cluster and the dissimilarity between the clusters (7, 8). This approach has recently been applied to make sense of heterogeneous assessment data in a range of mental health populations (9–12).

It is hypothesised that the known characteristics associated with the TRR service type (1) will be replicated. Additionally, it is hypothesised that CA will identify sub-groups of consumers consistent with the intuitive groupings described by staff in our qualitative study (e.g. presence/absence of substance use issues, symptomatic acuity, referral source (acute inpatient/non-acute inpatient) and accommodation issues. The impact of the integrated staffing model on service utilisation will also be explored. While it is hypothesised that similar consumers will access the services based on the shared service model, it is possible that the altered staffing configuration may impact the profile of consumers accepted into the service.

## Methods

### Ethical Clearance and Protocol Availability

This study presents cross-sectional data emerging from a parent prospective cohort study. The protocol for the parent study was developed following the STROBE statement (13) and published as a study in progress in June 2016 (2). 

### Study Context

The three CCUs under investigation are operated by a large public mental health service in Brisbane, the capital city of the state of Queensland in Australia. These CCUs are clinically operated public residential mental health services consistent with the TRR type defined in the typology by Parker et al. (1). The units operate under a shared model of service that focuses on the provision of transitional residential support to consumers aged 18–65 years who have a severe and persistent mental illness that substantially impairs their psychosocial functioning and capacity for independent living (14). The model of service designates the service as being recovery-oriented, and the nature of support described is consistent with the Australian National Framework for Recovery Oriented Mental Health Services (15).

Accommodation is provided in independent living units in a cluster-housing arrangement. The services are staffed 24-hours a day, with rehabilitation support focused on enhancing independent living skills (e.g. budgeting, cooking and cleaning) and community integration. Therapeutic interventions are also available on site including Cognitive Behaviour Therapy, Cognitive Remediation Therapy (16) and social cognitive interventions (17, 18). It is expected residents will be assisted to transition from the CCU to an alternative residence in the community after 6–24 months.

An “integrated staffing model” was being trialed at two of the three study sites. Under this staffing configuration peer support workers rather than nursing staff occupy the majority of staff roles (6). These peer support workers are employed based on their lived experience of mental issues and recovery, and actively contribute as a part of the multi-disciplinary team to the planning and delivery of rehabilitation support. This alternative staffing configuration was not intended to alter the core service model and rehabilitation function of the CCUs. Further details about the study sites are provided in **Table 1**.

**Table 1:** Details about the location, referring district, philosophy of care, physical environment, and staffing of the study sites.

			Site 1	Site 2	Site 3
**Location**		Distance from state capital (km)	4.2	30.9	21.2
		Socio-Economic Disadvantage, 2011* (19)	90	83	46
**Referring district**		Population (20)	588,475	143,628	287,517
		Acute inpatient services	Yes	Yes	Yes
		Community mental services	Yes	Yes	Yes
		Inpatient rehabilitation mental health beds	No	Yes	No
		Transitional housing team	Yes	No	No
		Outpatient community-based rehabilitation	Yes	No	Yes
		Mental health homelessness team	Yes	No	Yes
**CCU**	Philosophy of care	Recovery-oriented	Yes	Yes	Yes
		Strengths-based	Yes	Yes	Yes
		Designated rehabilitation focus	Yes	Yes	Yes
		Voluntary engagement in rehabilitation^	Yes	Yes	Yes
		Individualised care planning	Yes	Yes	Yes
		Transitional support	Yes	Yes	Yes
		Peer support role in care planning and delivery	Limited	Focused	Focused
	Physical environment	Maximum occupancy (consumers)	20	20	16
		Self-contained independent living units	20	20	15
		Disabled access units	1/20	1/20	1/15
		Shared recreation and leisure facilities	Yes	Yes	Yes
	Treatment & support	Individual psychotherapy support (CBT)	Yes	Yes	Yes
		Living skills support and development	Yes	Yes	Yes
		Structured leisure and physical activities	Yes	Yes	Yes
		Evidence-based therapeutic group programmes	Yes	Yes	Yes
	Staffing	Staffing model	Clinical	Integrated	Integrated
		Total FTE staff	21.5	24.5	18.4
		Total FTE peer-support staff	0.6	16	10.4
		Total FTE clinical staff	19.9	7.5	7
		Peer support: Clinical staff ratio	0.03	2.13	1.49
		Staff: Consumer ratio	1.08	1.23	1.15

*Local Government Area (LGA) percentile rank of the index of Relative Socio-Economic Disadvantage in comparison to all other LGAs in Australia, higher scores Equate to lower levels of disadvantage.

^Involuntary consumers are accepted at all three CCUs with explicit emphasis on voluntary engagement in available rehabilitation activities.

Consumer’s commencing at a CCU understand the nature of the service and have positive expectations of the experience including that of personal transformation (21, 22). Importantly, the way consumer hope to be treated in these settings is consistent with the principles of recovery-oriented care. Consumers describe several types of goals associated with service engagement, including independent living, getting a job, social re-integration and skills development, and improved health and fitness (22). While most consumers indicate they are actively involved in the decision to come to a CCU, issues of accommodation instability are a more commonly reported motivation than the availability of rehabilitation support (22).

### Participants and Data Collection

Recruitment occurred between December 2014 and December 2017. The commencement of recruitment coincided with the opening of the two sites operating the integrated staffing model. The site operating the clinical staffing model commenced operation in 2012, while the two integrated staffing models commenced operation in December 2014 and January 2015. A clinical assessment battery was completed with all consumers on service entry, including measures of direct relevance to the planning of individualized rehabilitation support. All consumers entering the CCU who remained beyond the 6-week initial assessment phase were eligible for inclusion in the study. A total of 145 out of the 161 (90%) consumers meeting the inclusion criterion provided voluntary informed consent for their data to be included in the study. Data was collected prospectively using a paper-based assessment battery by trained staff. The nature of the assessment and care delivered to consumers did not alter based on study participation.

The clinical assessment battery covered a broad range of domains relevant to the planning and evaluation of rehabilitation care (see **Table 2**). This battery was completed within the first 6-weeks of each consumer’s stay.

**Table 2:** Domains, focus, measures and raters of the initial clinical assessment battery.

Domain	Focus	ACL*	AC-QoL*	AUDIT*	BAS*	BPRS*^,#^	HONOS*	MHI-38*	LSP-16*	PRPP*	SANS*	SFS*	STORI-30*
**Behaviour**	Compliance	–	–	–	–	–	–	–	CL^^^	–	–	–	–
	Problematic	–	–	–	–	–	CL	–	–	–	–	–	–
	Resistance	–	–	–	–	CL	–	–	–	–	–	–	–
**Carer**	Carer burden	–	–	–	CR^^^	–	–	–	–	–	–	–	–
	Carer quality of life	–	CR	–	–	–	–	–	–	–	–	–	–
**Functioning**	Disability	–	–	–	–	–	–	–	CL	–	–	–	–
	Functioning (Task)	–	–	–	–	–	–	–	–	CL	–	–	–
	Global functioning	–	–	–	–	–	CL	–	–	–	–	–	–
	Social function	–	–	–	–	–	CL	–	–	–	–	CL	–
**Recovery**	Wellbeing	–	–	–	–	–	–	CO	–	–	–	–	–
	Recovery	CL	–	–	–	–	–	–	–	–	–	–	CO^^^
**Symptoms**	Cognition		–	–	–	–	CL	–	–	–	–	–	–
	Negative symptoms	–	–	–	–	CL	–	–	–	–	CL	–	–
	Positive symptoms	–	–	–	–	CL	–	–	–	–	–	–	–
	Distress	–	–	–	–	CL	CL	CO	–	–	–	–	–
	Substance use	–	–	CL/CO	–	–	CL	–	–	–	–	–	–

*Measures: Adult Carer Quality of Life (AC-QoL) (23), Alcohol Use Disorders Identification Test (AUDIT) (24), Allen’s Cognitive Levels (ACL) (25), Brief Psychiatric Rating Scale (BPRS) (26), Burden Assessment Scale (BAS) (27), Health of the Nation Outcome Scale (HoNOS) (28), Life Skills Profile (LSP-16) (29), Mental Health Inventory (MHI-38) (30), Perceive Recall Plan & Perform System of Task Analysis (PRPP) (31), Scale for the Assessment of Negative Symptoms (SANS) (32), Social Functioning Scale (SFS) (33), Stages of Recovery Instrument (STORI-30) (34).

^#^Factor structure for BPRS derived from Lachar et al. (26).

^Raters: Clinician-rated measure (CL), Consumer rated/self-report measure (CO), Carer rated measure (CR).

### Analyses

All analyses were completed in IBM SPSS Statistics Version 25. Statistical significance was assessed at an alpha value of 0.05. Where relevant, the Bonferroni correction was applied for multiple comparisons.

#### Cohort Description

Demographic, diagnostic, treatment-related, and assessment measures were analyzed using descriptive statistics. For the Brief Psychiatric Rating Scale (BPRS), the sub-scales derived from the factor analysis of Lachar et al. (26) were calculated.

#### Comparability of Included Sites

For dichotomous and nominal variables the comparability of the data collected from the three included sites was assessed using chi-square or Fisher’s exact test when the expected cell count for any cell was <5 (35). Where significant differences on contingency tables exceeding 2 × 2 were found, adjusted standardized residuals were assessed to identify cells having a statistically significant difference between the observed and expected frequencies (36). For significant differences identified through chi-square analyses, the contribution of individual cells was examined using the +/-2 criteria for adjusted standardized residuals (37).

For continuous and scaled variables, normality was assessed using the Shapiro-Wilks W test, and homogeneity of variance was assessed using Levene’s test. ANOVA was used for variables not violating these assumptions. For variables violating the assumption of normality the Kruskal-Wallis statistic was calculated. If marked differences in sample size from the study sites emerged and the assumption of homogeneity of variance was violated the use of Welch’s ANOVA was planned to be considered. Analyses were also repeated using only the subset of consumers diagnosed with schizophrenia spectrum disorders (F20-29.x); these analyses are presented in the **Supplementary Materials**.

#### Comparison With the Pooled Transitional Residential Rehabilitation Cohort

The equivalence of the cohort with the available data from a pooled TRR cohort generated through a previous systematic review (1) was evaluated using independent samples chi-square/Fisher’s exact test for categorical variables. Statistical comparison of continuous variables was unable to be performed due to inconsistently reported standard deviations in the studies included in the TRR cohort. The contribution of other studies associated with sub-samples derived from the cohort generated in the present study (n = 24) (20) was removed from the pooled TRR cohort before comparisons occurred. Only variables with data available from ≥50% of the total cases in the TRR cohort were considered.

#### Approach to Missing Data

Patterns of missing data in the assessment battery were explored using the SPSSv25 Missing Values Analysis module. Levels of missing data are detailed in the Results section, and variables with ≥50% missing data were excluded. The acceptability of the assumption that data was at least missing at random was considered based on the total scores for included measures using Little’s MCAR test.

#### Cluster Analysis

The CA was run on complete cases using a reduced set of clinician-rated variables where the level of missing data did not exceed 80%. A two-stage approach was taken with a view to achieving an optimal clustering algorithm (7, 38, 39):

1. Hierarchical CA using Wards methods of minimum variance based on squared Euclidean distance was conducted to identify an optimal number of clusters. The optimal number of clusters was determined based on examination of the magnitude of change of in the coefficients on the agglomeration schedule, and verified *via* examination of the scree plot (“elbow method”) as well as inspection of the dendrogram.
2. The hierarchical CA was repeated using k-means clustering to segregate the cohort into the optimal number of clusters defined at Stage 1.

The reliability of the cluster solution was evaluated through examination of the stability of cluster membership on re-assignment using two methods suggested in the literature (40): k-means clustering of a randomly selected 50% sample of cases, and with randomization of case order. Confirmatory (standard) discriminant function analysis was used to establish which clinician-rated variables best distinguished the cluster groups; variables were considered important contributors if the coefficient was ≤-.3 or ≥.3 (41). Differences in the demographic, diagnostic and assessment profile of the identified cluster sub-groups were assessed using an identical analytic approach to that outlined under the sub-heading “Comparability of Included Sites”.

## Results

### Comprehensive Cohort Description

The admission cohort included 145 consumers. Complete data was available for all demographic (**Table 3**), diagnostic (**Table 4**) and treatment-related variables (**Table 5**). Due to extensive missing data in the carer-rated measures [ACQoL (71.7%) and BAS (69.7%)], these measures were omitted from the analysis. The proportion of missing data in the assessment battery was less than 10% for all clinician and consumer-rated measures, except for the PRPP (23.4%). Following exclusion of the carer-rated measures: 40% of cases had missing data in the assessment battery; the overall proportion of missing data was 7.3%; and the acceptability of the assumption that data was missing at random was supported (X^2^_(128)_ = 154.006, p=.058). Clinician-rated measures are summarized in **Table 6**, and consumer-rated measures are summarized in **Table 7**. Additional information is available in the **Supplementary Materials**, including *post hoc* comparisons, and sub-analyses limited to the F20-29.x diagnostic grouping.

**Table 3:** Demographics of the CCU admission cohort.

Staffing model	Clinical	Integrated		Total	Test^e^	p
Site	Site 1 (n = 53)	Site 2 (n = 52)	Site 3 (n = 40)	N = 145		
**Demographics**						
Age at admission ($\bar{\text{x}}$, years)	31.1 (8.7)	32.1 (8.7)	31.0 (9.8)	31.4 (9.0)	F_(2,142)_ = **.**214	.808
Male sex	66.0%	78.8%	77.5%	73.8%	X^2^_(2)_ = 2.619	.270
Australian born	86.8%	90.4%	77.5%	85.5%	X^2^_(2)_ = 3.140	.208
ATSI identification	6.0%	3.8%	10%	6.2%	Fisher’s Exact Test^f^	.525
Unemployment^a^	90.6%	82.7%	95.0%	89.0%	X^2^_(2)_ = 3.707	.157
**Accommodation (most recent)** ^b^					Fisher’s Exact Test^f^	.066
Living with family	56.6%	50.0%	72.5%	58.6%	–	–
Supported housing	18.9%	5.8%	10.0%	11.7%	–	–
Private rental	9.4%	15.4%	10.0%	11.7%	–	–
No fixed address	7.5%	21.2%	2.5%	11.0%	–	–
Other	7.5%	7.7%	5.0%	6.9%	–	–
**Highest education level** ^c^					H_(2)_ **=** 1.898	.387
Primary school	5.7%	3.8%	7.5%	5.5%	–	–
Year 10	41.5%	55.8%	50.0%	49.0%	–	–
Year 12	34.0%	19.2%	35.0%	29.0%	–	–
Tertiary^d^	18.9%	19.2%	7.5%	15.9%	–	–

a Unemployment is exclusive of any form of paid or unpaid vocational activity including volunteering.

b Accommodation reflects the most recent community residence prior to CCU entry, public housing accounted for 70% of the ‘Other’ category.

c Treated as a scaled variable based on increasing levels of education, Kruskall-Wallis test applied.

d Inclusive of any engagement in tertiary education including vocational training regardless of completion

e For categorical variables, X^2^ was applied unless the expected count for any cell was <5, in this case, Fisher’s Exact test was calculated.

f Unadjusted odds ratio: Accommodation *=* 14.200, ATSI identification *=* 1.500.

**Table 4:** Primary diagnosis and co-morbidity data for CCU Admission cohort.

Staffing model	Clinical	Integrated		Total	Test^b^	p
Site	Site 1 (n = 53)	Site 2 (n = 52)	Site 3 (n = 40)	N = 145		
**Primary diagnosis**	****	****	****	****	****	****
F20-29.x Schizophrenia spectrum	71.7%	73.1%	90.0%	77.2%	X^2^_(2)_ = 5.143	.076
Specific disorders^a^:						
- F20.x Schizophrenia	47.2%	65.4%	67.5%	59.3%	–	–
- F25.x Schizoaffective disorder	18.9%	5.8%	17.5%	13.8%	–	–
- F29.x Unspecified psychosis	5.7%	1.9%	5.0%	4.1%	–	–
- F31.x Bipolar disorder	15.1%	11.5%	2.5%	10.3%	–	–
- F32-34.x Depressive disorders	5.7%	11.5%	5.0%	7.6%	–	–
- Other disorders	7.4%	3.9%	2.5%	4.9%	–	–
**Secondary diagnoses/issues**	****	****	****	****	****	****
Current tobacco use	30.2%	73.1%	70.0%	56.6%	X^2^_(2)_ = 23.715	.000
Substance use	37.7%	53.8%	42.5%	44.8%	X^2^_(2)_ = 2.875	.237
Physical health issue	22.6%	17.3%	35.0%	24.1%	X^2^_(2)_ = 3.967	.138
Trauma history	9.4%	11.5%	7.5%	9.7%	Fisher’s Exact Test^c^	.883
Anxiety disorder	11.3%	9.6%	2.5%	8.3%	Fisher’s Exact Test^c^	.290
Developmental disorder	7.5%	9.6%	7.5%	8.3%	Fisher’s Exact Test^c^	.932
Personality disorder	5.7%	9.6%	5.0%	6.9%	Fisher’s Exact Test^c^	.711
Obsessive-Compulsive Disorder	1.9%	9.6%	2.5%	4.8%	Fisher’s Exact Test^c^	.152

a Test statistic calculated only for the presence/absence of F20-29.x diagnoses (see above) given the number of diagnostic categories.

b For categorical variables, the Chi-Square test was applied unless the expected count for any cell was <5, in this case, Fisher’s Exact test was calculated.

c Unadjusted odds ratio: Trauma history = 0.445, Anxiety disorder = 0.256, Developmental disorder = 0.266, Personality Disorder = 0.890, Obsessive-Compulsive Disorder = 3.321.

**Table 5:** Treatment-related information for the CCU admission cohort.

Staffing model	Clinical	Integrated		Total	Test	p
	Site 1 (n = 53)	Site 2 (n = 52)	Site 3 (n = 40)	(N = 145)		
Referral and Legal status						
Community-based referral^a^	56.6%	63.5%	62.5%	60.7%	X^2^_(2)_ = .593	.743
Involuntary treatment^b^	52.8%	51.9%	32.5%	46.9%	X^2^_(2)_ = .4.606	.102
Guardianship order present	5.7%	3.8%	5.0%	4.8%	Fisher’s Exact Test^c^	1.000
**Medications prescribed**						
Anti-psychotic medication:						
- CPZ equivalent dose ($\bar{\text{x}}$, mg)	436.2 (365.3)	436.4 (284.5)	361.3 (257.7)	415.6 (309.8)	K_(2)_ = 2.073	.355
- Depot prescribed	45.3%	50.0%	40.0%	45.5%	X^2^_(2)_ = 0.914	.633
- Clozapine prescribed	17.0%	25.0%	37.5%	25.5%	X^2^_(2)_ = 5.061	.080
- Number of antipsychotics	1.36 (0.71)	1.42 (0.696)	1.15 (0.58)	1.32 (0.676)	K_(2)_ = 4.528	.104
Mood stabiliser:						
- Lithium	20.8%	21.2%	10.0%	17.9%	X^2^_(2)_ = 2.364	.307
- Sodium valproate	9.4%	15.4%	12.5%	12.4%	X^2^_(2)_ = .855	.652
- Other	7.5%	3.8%	0.0%	4.1%	X^2^_(2)_ = 3.291	.193
Other medication:						
- Antidepressant	41.5%	44.2%	42.5%	42.8%	X^2^_(2)_ = .081	.960
- Benzodiazepine(s)	13.2%	17.3%	7.5%	13.1%	X^2^_(2)_ = 1.911	.385

a Community-based referral compared to combined acute (35.2%) and sub-acute (4.1%) inpatient referral source.

b Involuntary treatment includes both Involuntary Treatment Orders (43.5%) and Forensic Orders (3.4%).

c Unadjusted odds ratio: Guardianship order present = .359.

**Table 6:** Clinical assessment battery for the CCU admission cohort, mean scores and standard deviation.

Staffing model	Clinical		Integrated				N^a^	Total		
	Site 1		Site 2		Site 3					
	n	$\bar{\text{x}}$(SD)	n	$\bar{\text{x}}$(SD)	n	$\bar{\text{x}}$(SD)		$\bar{\text{x}}$(SD)	Test	p
**Functioning and disability**										
HoNOS (Total)	53	8.98(6.125)	51	9.75(4.707)	40	12.80(6.638)	144	10.31(5.992)	K_(2)_ = 9.444	.009^b^
- Behaviour		1.13(1.699)		1.06(1.475)		1.20(1.682)		1.13(1.608)	K_(2)_ = .169	.919
- Impairment		1.38(1.431)		1.49(1.317)		2.48(1.633)		1.72(1.517)	K_(2)_ = 13.293	.001^c^
- Symptoms		2.74(2.159)		3.65(2.331)		4.55(2.501)		3.56(2.414)	K_(2)_ = 12.303	.002^b^
- Social		3.74(2.995)		3.55(2.766)		4.58(3.161)		3.90(2.974)	K_(2)_ = 2.740	.254
LSP-16 (Total)	50	10.78(5.643)	51	12.53(6.166)	39	13.62(5.775)	140	12.21(5.945)	K_(2)_ = 5.262	.072
- Withdrawal		2.58(1.864)		2.92(1.864)		3.26(1.860)		2.89(1.869)	K_(2)_ = 4.042	.132
- Self-care		3.38(2.118)		4.53(2.411)		5.38(2.208)		4.36(2.378)	K_(2)_ = 16.799	.000^b^
- Compliance		2.14(1.539)		2.10(1.652)		1.77(1.512)		2.02(1.571)	K_(2)_ = 1.036	.596
- Anti-social		1.48(1.515)		1.43(1.723)		1.46(1.620)		1.46(1.611)	K_(2)_ = .212	.899
Allen Cognitive Level	51	5.03 (.405)	48	5.16(.4261)	40	5.01(337)	139	5.07(.398)	K_(2)_ = 5.345	.069
Social Functioning Scale	51	107.05(7.814)	50	102.95(7.996)	39	100.84(7.784)	140	103.85(8.224)	K_(2)_ = 13.362	.001^d^
**Symptomatic measures**										
BPRS-18 (Total)	51	37.47(8.889)	46	36.67(9.778)	36	42.81(9.730)	133	38.64(9.707)	K_(2)_ = 8.162	.017^e^
- Resistance		6.08(2.606)		5.89(1.816)		5.92(2.285)		5.97(2.256)	K_(2)_ = .290	.865
- Positive symptoms		10.51(5.108)		10.33(4.634)		12.25(5.369)		10.92(5.054)	K_(2)_ = 3.337	.189
- Negative symptoms		6.76(3.664)		6.02(3.363)		7.69(3.060)		6.76(3.442)	K_(2)_ = 6.790	.034^e^
- Psychological discomfort		13.00(4.152)		13.09(5.001)		15.50(5.364)		13.71(4.888)	F_(2,130)_ = 3.449	.035^f^
SANS (Total)	51	43.53(18.884)	49	49.53(16.686)	36	50.61(18.243)	136	47.57(18.094)	F_(2,133)_ = 2.102	.126
- Affective flattening		14.65(8.756)		14.90(8.295)		14.94(8.349)		14.77(8.442)	K_(2)_ = .122	.941
- Alogia		3.29(4.125)		4.86(4.168)		5.58(4.129)		4.53(4.267)	K_(2)_ = 10.231	.006^d^
- Avolition/apathy		8.71(4.494)		10.27(2.782)		10.64(3.331)		9.78(3.699)	K_(2)_ = 8.643	.013^b^
- Anhedonia/asociality		13.41(5.193)		15.08(3.834)		14.69(4.125)		14.29(4.553)	F_(2,133)_ = 1.892	.155
- Attention		3.47(3.349)		4.43(3.482)		4.75(2.802)		4.17(.374)	K_(2)_ = 4.564	.102
**Substance use (alcohol)**										
AUDIT	48	4.90(7.856)	50	10.38(10.111)	35	6.80(6.957)	133	7.46(8.839)	K_(2)_ = 12.809	.002^g^

a Available sample size varies based on missing data: HoNOS (.9%), LSP-16 (3.5%), Allens Cognitive Level (4.1%), SFS (3.4%), BPRS-18 (8.3%), SANS (6.2%) and AUDIT (8.3%).

b Post-hoc tests with Bonferroni correction for multiple tests identified statistically significant pairwise comparison between Sites 1 and 3 only.

c Post-hoc tests with Bonferroni correction for multiple tests identified statistically significant pairwise comparison between Site 3 and Sites 1&2.

d Post-hoc tests with Bonferroni correction for multiple tests identified statistically significant pairwise comparison between Site 1 and Sites 2&3.

e Post-hoc tests with Bonferroni correction for multiple tests identified statistically significant pairwise comparison between Sites 2 and 3 only.

f Post-hoc tests with Bonferroni correction for multiple tests identified no statistically significant pairwise comparisons.

g Post-hoc tests with Bonferroni correction for multiple tests identified statistically significant pairwise comparison between Sites 1 and 2 only.

**Table 7:** Consumer rated assessments for the CCU admission cohort.

	Clinical staffing		Integrated staffing				Total			
	Site 1		Site 2		Site 3					
	n	$\bar{\text{x}}$(SD)	n	$\bar{\text{x}}$(SD)	n	$\bar{\text{x}}$(SD)	N^a^	$\bar{\text{x}}$(SD)	Test	p
**Mental Health Inventory (Total)**	52	57.52(17.076)	52	53.17(21.787)	40	56.40(21.358)	144	55.64(20.034)	K_(2)_ = 1.899	.387
Psychological wellbeing		47.50(21.775)		41.92(25.469)		47.08(19.737)		45.37(22.660)	K_(2)_ = 1.488	.475
Psychological distress		34.04(21.718)		38.90(24.579)		32.60(24.674)		35.40(23.602)	K_(2)_ = 1.385	.500
**STORI-30**	45	–	47	–	40	–	132	–	Fisher’s exact^b^	.318
Moratorium	7	15.6%	5	10.6%	2	5.0%	14	10.6%		
Awareness	17	37.8%	10	21.3%	18	45.0%	45	34.1%		
Preparation	3	6.7%	3	6.4%	3	7.5%	9	6.8%		
Rebuilding	7	15.6%	13	27.7%	6	15.0%	26	19.7%		
Growth	11	24.4%	16	34.0%	11	27.5%	38	28.8%		

a Available sample size varies based on missing data: MHI (.6%), STORI-30 (9.0%).

b Unadjusted odds ratio: STORI-30 = 9.228.

### Comparability of Included Sites

No statistically significant differences emerged in the study sites for demographic and treatment-related variables. The only difference between study sites with regards to diagnostic variables was the likelihood of being a current smoker at the time of admission. Consumers admitted to Site 1 (clinical staffing, adjusted residual -4.860) were less frequently current smokers than those at the two integrated staffing model sites (Sites 2 and 3, adjusted residuals 3.002 and 2.016, respectively).

Statistically significant differences emerged in the total measure scores on the clinical assessment battery for Health of the Nation Outcome Scale (HoNOS), Social Functioning Scale (SFS), and Alcohol Use Disorders Identification Test (AUDIT); but not for Life Skills Profile (LSP-16), Allen’s Cognitive Levels (ACL), or Scale for the Assessment of Negative Symptoms (SANS). Total HoNOS was higher at Site 1 (clinical staffing) than Site 3, indicating better mental health and social functioning. Total SFS was lower at Site 1 (clinical staffing) than Sites 2 & 3 indicating better social functioning. Total BPRS was lower at Site 2 than Site 3, indicating lower levels of psychiatric symptoms at this site. Total AUDIT was lower at Site 1 than Site 2, indicating lower levels of problematic alcohol use at Site 1.

### Comparability With the TRR-Cohort Presented in Parker et al.

Full details of the comparisons between our cohort and the modified TRR cohort are provided in the **Supplementary Materials**. Statistical comparison between our sample and the modified TRR cohort found no significant differences in the distribution of demographic variables, including male sex (73.8% versus 72.5%, X^2^_(2)_ = 0.106, p = .744), Australian-birth (85.5% versus 85.0%, X^2^_(2)_ = 0.030, p = .863), and identification as an Aboriginal and/or Torres Strait Islander (ATSI, 6.2% versus 9.3%, X^2^_(2)_ = 1.455, p = .228). The absence of standard deviation data prevented statistical comparison of the weighted mean age in the modified TRR cohort and our sample (35.5 and 31.4 years, respectively). The frequency of being subject to a guardianship order was lower in our sample than the modified TRR cohort (4.8% versus 42%, X^2^_(2)_ = 71.61, p = .000). No significant differences on other available treatment-related variables were identified between our cohort and the modified TRR cohort: community-based referral (60.7% versus 55.7%, X^2^_(2)_ = 1.199, p = .274) and involuntary treatment (46.9% versus 49.1%, X^2^_(2)_ = .255, p = .614).

A primary diagnosis of F20-29.x disorders occurred less frequently in our cohort than the modified TRR cohort (77.2% versus 86.2%, X^2^_(2)_ = 8.046, p = .005). Substance use issues occurred more frequently in our sample than in the modified TRR cohort (44.8% versus 20.5%, X^2^_(2)_ = 40.469, p = .000), and physical health issues were identified less commonly (24.1% versus 36.4%, X^2^_(2)_ = 8.499, p = .004).

### Cluster Analysis

The CA was performed on the 111 cases (76.6%) that had complete data for clinician-rated assessments excluding the PRPP. Hierarchical CA using Ward’s Method identified three as the optimal number of clusters to be evaluated based on the visually assessed demarcation point of agglomeration coefficients via scree plot. Hierarchical CA was then re-run using the K-means method to allocate cases across 3 clusters optimally. The cluster solution distributed 17 cases to Cluster 1, 43 cases to Cluster 2 and 51 cases to Cluster 3. The reliability of this solution was supported by identical re-allocation of 91% of cases following case order randomization and 73% of cases when a random sample of ∼50% of cases was analyzed.

**Figure 1:**
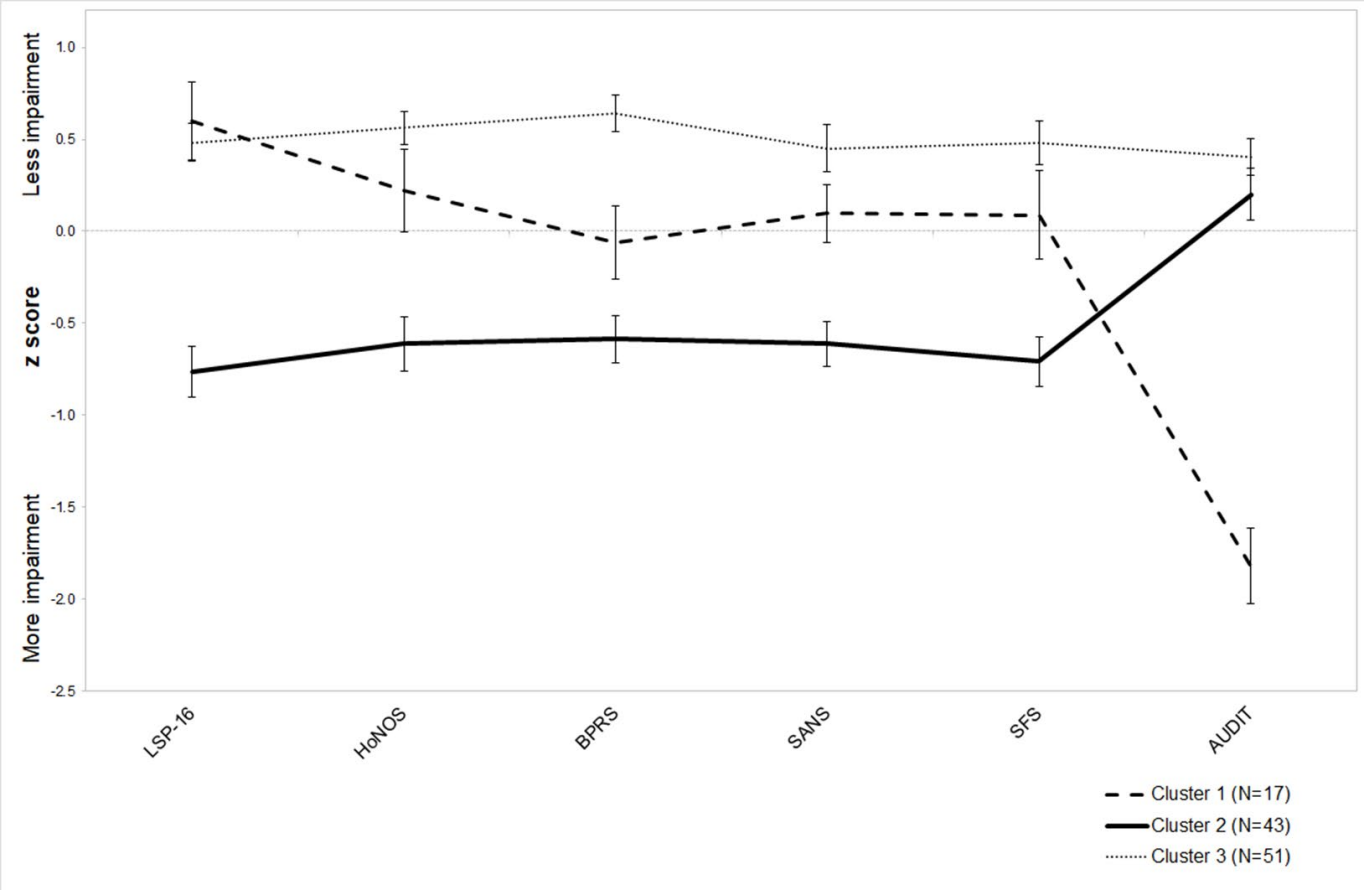
Final cluster solution with z-score means and standard error by cluster for variables making a significant contribution to the underlying factors.

Confirmatory discriminant function analysis identified two functions (Λ_14_.378, p < .000): Function 1 accounting for 59.6% of the variance, and Function 2, accounting for 40.5% of the variance. The structure matrix indicated that the variables making important contributions to discrimination between clusters were: LSP-16, HoNOS, BPRS, SFS, and SANS (Function 1: .489, .405, .401, .381, and .353 respectively); and AUDIT (Function 2: .911). **Figure 1** presents the z-score means and standard errors for these discriminating variables. Cluster 1 allocation infrequently occurred (15% of the sample) and was characterized by higher levels of alcohol use (AUDIT) than the other clusters. Cluster 2 (39% of the sample) was characterized by higher levels of disability (LSP-16), lower levels of mental health (HoNOS) and social function (HoNOS and LSP), and higher levels of negative psychotic symptoms (SANS) than the other clusters. Cluster 3 predominated (46% of the sample) and was characterized lower levels of general psychiatric symptoms (BPRS) than the other clusters.

No significant differences emerged between the clusters on the demographic or treatment-related variables (**Tables 8**, **9**). The only significant differences between the clusters on diagnostic variables were the increased likelihood of co-morbid substance use issues (X^2^_(2)_ = 21.240, p < .000, adjusted residual = 4.6) and co-morbid personality disorder/traits (Fisher’s exact test p < .042, adjusted residual = 2.8) for participants assigned to Cluster 1 (**Table 10**). Patterns of sub-scale data for the clinician-rated variables were generally consistent with the findings based on the total scores (**Table 11**).

**Table 8:** Demographics by cluster.

Site	Cluster 1 (n = 17)	Cluster 2 (n = 43)	Cluster 3 (n = 51)	TOTAL (N = 111)		
Demographics						
Age at admission ($\bar{\text{x}}$, years)	31.35(7.441)	32.98(10.809)	29.82(8.294)	31.28(9.276)	F_(2,108)_ = 1.357	.262
Male sex	76.5%	79.1%	70.6%	74.8%	Fisher’s Exact Test	.669
Australian born	88.2%	90.7%	86.3%	88.3%	Fisher’s Exact Test	.857
ATSI identification	17.6%	4.9%	6.3%	7.2%	Fisher’s Exact Test	.178
Unemployment^a^	82.4%	97.7%	90.2%	91.9%	Fisher’s Exact Test	.090
**Accommodation (most recent)**					Fisher’s Exact Test	.133
Living with family	58.8%	55.8%	62.7%	59.5%	–	–
Supported housing	5.9%	20.9%	5.9%	11.7%	–	–
Private rental	23.5%	7.0%	9.8%	10.8%	–	–
No fixed address	5.9%	7.0%	17.6%	11.7%	–	–
Other^b^	5.9%	9.3%	3.9%	6.3%	–	–
**Highest education level** ^c^					H_(2)_ = 3.538	.171
Primary school	5.9%	4.7%	7.8%	6.3%	–	–
Year 10	52.9%	55.8%	35.3%	45.9%	–	–
Year 12	35.3%	25.6%	35.3%	31.5%	–	–
Tertiary^d^	5.9%	14.0%	21.6%	16.2%	–	–

a Unemployment is exclusive of any form of paid or unpaid vocational activity including volunteering.

b Public housing accounts for 70% of the ‘Other’ category.

c Treated as a scaled variable based on increasing levels of education, Kruskal-Wallis test applied.

d Inclusive of any engagement in tertiary education including vocational training regardless of completion.

**Table 9:** Treatment-related variables by cluster.

	Cluster 1 (n = 17)	Cluster 2 (n = 43)	Cluster 3 (n = 51)	TOTAL (N = 111)	Test^b^	p
Referral and Legal status						
Community-based referral	52.9%	69.8%	62.7%	64.0%	X^2^_(2)_ = 1.557	.459
Involuntary treatment^b^	41.2%	41.9%	41.2%	41.4%	X^2^_(2)_ = .005	.997
Guardianship order present	–	2.3%	7.8%	4.5%	Fisher’s Exact Test^c^	.459
**Medications prescribed**						
Anti-psychotic medication:						
- CPZ equivalence, mg ($\bar{\text{x}}$, SD)	522.2(359.7)	391.5(212.1)	382.6(322.7)	407.41(292.9)	K_(2)_ = 2.373	.305
- Depot prescribed	52.9%	46.5%	43.1%	45.9%	X^2^_(2)_ = .502	.778
- Clozapine prescribed	29.4%	16.3%	27.5%	23.4%	Fisher’s Exact Test^c^	.336
- Number of antipsychotics	1.41(.618)	1.28(.630)	1.31(.678)	1.32(.676)	K_(2)_ = .756	.685
Mood stabiliser:						
- Lithium	23.5%	16.3%	5.9%	14.4%	Fisher’s Exact Test^c^	.506
- Sodium valproate	11.8%	16.3%	5.9%	10.8%	Fisher’s Exact Test^c^	.291
- Other	–	2.3%	7.8%	4.5%	Fisher’s Exact Test^c^	.459
Other medication:						
- Antidepressant	29.4%	51.2%	37.3%	41.4%	X^2^_(2)_ = 3.057	.217
- Benzodiazepine(s)	17.6%	16.3%	5.9%	11.7%	Fisher’s Exact Test^c^	.174

a Community-based referral compared to combined acute and sub-acute inpatient referral source.

b Involuntary treatment includes both Involuntary Treatment Orders and Forensic Order.

c Unadjusted odds ratio: Guardianship order present = 1.758, Clozapine prescribed *=* 2.139; Lithium prescribed *=* 1.545; Sodium valproate prescribed *=* 2.718; Other mood stabiliser prescribed *=* 1.758; Benzodiazepine(s) *=* 3.419.

**Table 10:** Diagnosis by cluster.

	Cluster 1 (n = 17)	Cluster 2 (n = 43)	Cluster 3 (n = 51)	TOTAL (N = 111)	Test^b^	p
Primary diagnosis^a^						
F20-29.x Schizophrenia spectrum	82.4%	88.4%	72.5%	80.2%	Fisher’s Exact Test^c^	.156
Specific disorders^a^:						
- F20.x Schizophrenia	64.7%	60.5%	64.7%	63.1%	–	–
- F25.x Schizoaffective disorder	17.6%	20.9%	3.9%	12.6%	–	–
- F29.x Unspecified psychosis	–	7.0%	3.9%	4.5%	–	–
- F31.x Bipolar disorder	11.8%	2.3%	13.8%	9.0%	–	–
- F32-34.x Depressive disorders	5.9%	7.0%	5.9%	6.3%	–	–
- Other disorders	–	2.3%	3.9%	2.7%	–	–
**Secondary diagnoses/issues**						
Current tobacco use	70.6%	65.1%	47.1%	57.7%	X^2^_(2)_ = 4.491	.106
Substance use	94.1%	32.6%	35.3%	43.2%	X^2^_(2)_ = 21.240	.000^d^
Physical health issue	11.8%	27.9%	17.6%	20.7%	Fisher’s Exact Test^c^	.353
Trauma history	5.9%	2.3%	11.8%	7.2%	Fisher’s Exact Test^c^	.207
Anxiety disorder	5.9%	4.7%	15.7%	9.9%	Fisher’s Exact Test^c^	.191
Developmental disorder	5.9%	4.7%	13.3%	8.1%	Fisher’s Exact Test^c^	.456
Personality disorder	23.5%	4.7%	3.9%	7.2%	Fisher’s Exact Test^c^	.042^e^
Obsessive-Compulsive Disorder	–	9.3%	3.9%	5.4%	Fisher’s Exact Test^c^	.447

a Test statistic calculated only for the presence/absence of F20-29.x diagnoses (see above) given the number of diagnostic categories

b For categorical variables, the Chi Square test was applied unless the expected count for any cell was <5, in this case, Fisher’s Exact test was calculated

c Unadjusted odds ratio: F20-29.x Schizophrenia spectrum *=* 3.628, Substance use *=* 22.60, Physical health issue *=* 2.239; Trauma history *=* 2.943; Trauma history *=* 3.099; Developmental disorder *=* 1.513; Personality disorder *=* 6.082; Obsessive-Compulsive Disorder *=* 1.787

d Cells with adjusted standardised residuals ≥+2 *=* Cluster 1 (Substance use issue – Yes)

e Cells with adjusted standardised residuals ≥+2 *=* Cluster 1 (Personality Disorder – Yes)

**Table 11:** Clinician-rated measures and sub-scales by cluster*.

	Cluster 1 (n = 17)		Cluster 2 (n = 43)		Cluster 3 (n = 51)		Total (N = 111)		Test	p
	$\bar{\text{x}}$(SD)		$\bar{\text{x}}$(SD)		$\bar{\text{x}}$(SD)		$\bar{\text{x}}$(SD)			
Functioning and disability										
HoNOS (Total)	9.00	5.534	13.98	5.755	6.94	3.906	9.98	5.891	K_(2)_ = 35.674	.000^a^
- Behaviour	1.88	1.409	1.21	1.567	.51	1.255	.99	1.480	K_(2)_ = 21.306	.000^b^
- Impairment	1.41	1.734	2.40	1.482	1.10	1.237	1.65	1.529	K_(2)_ = 18.997	.000^a^
- Symptoms	3.65	2.548	4.84	2.468	2.41	1.878	3.54	2.475	K_(2)_ = 21.968	.000^c^
- Social	2.06	2.384	5.53	3.150	2.92	2.162	3.80	2.957	K_(2)_ = 24.865	.000^a^
LSP-16 (Total)	8.65	5.219	16.74	5.416	9.33	4.339	12.21	5.945	K_(2)_ = 40.508	.000^a^
- Withdrawal	1.53	1.218	4.37	1.865	2.18	1.352	2.92	1.882	K_(2)_ = 41.562	.000^a^
- Self-care	3.59	2.717	5.81	2.119	3.31	1.715	4.40	2.389	K_(2)_ = 30.522	.000^a^
- Compliance	1.47	1.625	2.49	1.549	1.65	1.494	2.06	1.603	K_(2)_ = 8.697	.013^c^
- Anti-social	1.18	1.237	1.84	1.717	1.18	1.545	1.45	1.610	K_(2)_ = 4.711	.095
Allen Cognitive Level	4.95	.445	5.00	.389	5.13	.407	5.054	.409	K_(2)_ = 3.714	.156
Social Functioning Scale	104.57	8.183	98.02	7.138	107.82	6.95	103.53	8.469	K_(2)_ = 34.695	.000^a^
**Symptomatic measures**										
BPRS-18 (Total)	39.24	8.066	44.33	8.225	32.41	6.885	38.07	9.338	K_(2)_ = 38.473	.000^b^
- Resistance	5.76	1.954	6.67	2.476	5.04	1.536	5.78	2.129	K_(2)_ = 13.685	.001^c^
- Positive symptoms	11.29	4.089	12.12	5.399	9.08	3.893	10.59	4.743	K_(2)_ = 9.232	.010^c^
- Negative symptoms	6.24	2.728	8.63	3.599	5.22	2.648	6.69	3.424	K_(2)_ = 22.763	.000^c^
- Psychological discomfort	14.94	4.220	15.30	5.040	11.98	4.474	13.72	4.899	F_(2,108)_ = 6.595	.002^c^
SANS (Total)	45.82	11.706	58.63	14.635	39.41	16.755	47.84	17.561	F_(2,108)_ = 18.616	.000^d^
- Affective flattening	14.18	7.427	18.67	7.177	11.63	7.997	14.75	8.207	K_(2)_ = 16.675	.000^c^
- Alogia	3.65	3.040	6.53	4.677	3.24	3.479	4.58	4.203	K_(2)_ = 14.393	.001^c^
- Avolition/apathy	9.76	2.818	11.88	2.312	8.47	3.797	9.99	3.497	K_(2)_ = 25.374	.000^a^
- Anhedonia/asociality	13.88	3.295	16.79	3.433	12.61	4.618	14.42	4.420	K_(2)_ = 21.703	.000^a^
- Attention	4.35	3.081	4.74	3.430	3.47	2.976	4.10	3.202	K_(2)_ = 3.383	.184
**Substance use (alcohol)**										
AUDIT	23.53	6.983	5.67	6.171	3.88	4.48	7.59	8.823	K_(2)_ = 42.393	.000^e^

*Differences between clinician-rated measures are a product of the cluster analysis and should not be used to infer true differences between groups (given that these differences provided the basis for group separation). This table is included to illustrate the contribution of sub-scales on which total scores are based to the cluster solution.

a Post-hoc tests with Bonferroni correction for multiple tests identified statistically significant pairwise comparison between Cluster 2 and 1&3.

b Post-hoc tests with Bonferroni correction for multiple tests identified statistically significant pairwise comparison between Cluster 3 and 1&2.

c Post-hoc tests with Bonferroni correction for multiple tests identified statistically significant pairwise comparison between Cluster 2 and 3 only.

d Post-hoc tests with Bonferroni correction for multiple tests identified statistically significant pairwise comparison between Cluster 1 and 2 (p *=* .013), and Cluster 2 and 3 (p *=* .000).

e Post-hoc tests with Bonferroni correction for multiple tests identified statistically significant pairwise comparison between Cluster 1 and 2&3.

Significant differences between the clusters emerged for the consumer-rated assessments (**Table 12**). Consumers allocated to Cluster 3 scored higher (more favorably) on the MHI-38 (K_(2)_ = 10.445, p = .005, pairwise comparisons between Cluster 1 and 2 were both statistically significant), this difference being driven by higher ratings on the psychological wellbeing sub-scale (K_(2)_ = 11.118, p = .004, pairwise comparisons between Cluster 1 and 2 were both statistically significant). Differences in the likelihood of allocation to various stages of recovery (STORI-30) emerged (Fisher’s exact test = .015, unadjusted odds ratio 17.810). These differences were accounted for by the increased likelihood of being in the “moratorium” phase and reduced likelihood of “growth” phase for Cluster 2 (adjusted standardized residuals 2.0 and -2.5 respectively), and reduced likelihood of being in the “moratorium” phase and increased likelihood of being in the “growth” phase for Cluster 3 members (adjusted standardized residuals -2.5 and 3.3 respectively).

**Table 12:** Consumer-rated measures by cluster.

	Cluster 1		Cluster 2		Cluster 3		N^a^	Insert inline(SD)	Test	p
	n	$\bar{\text{x}}$(SD)	n	$\bar{\text{x}}$(SD)	n	$\bar{\text{x}}$(SD)				
**MHI-38 (Total)**	17	49.88(22.209)	43	51.84(19.848)	51	63.00(17.034)	111	56.67(19.720)	K_(2)_ = 10.445	.005^a^
Psychological wellbeing		38.53(23.492)		40.00(22.018)		53.31(21.575)		45.89(22.901)	K_(2)_ = 11.118	.004^a^
Psychological distress		43.41(24.308)		39.26(22.065)		27.12(22.230)		34.32(23.298)	K_(2)_ = 7.836	.020^b^
**STORI-30**	16	–	42	–	47	–	105	–	Fisher’s Exact Test^b^	.015^c^
Moratorium	3	18.8%	9	21.4%	2	4.3%	14	13.3%		
Awareness	7	43.8%	15	35.7%	10	21.3%	32	30.5%		
Preparation	1	6.3%	2	4.8%	6	12.8%	9	8.6%		
Rebuilding	2	12.5%	9	21.4%	7	14.9%	18	17.1%		
Growth	3	18.8%	7	16.7%	22	46.8%	32	30.5%		

a Post-hoc tests with Bonferroni correction for multiple tests identified statistically significant pairwise comparison between Cluster 3 and 1&2

b Post-hoc tests with Bonferroni correction for multiple tests identified no statistically significant pairwise comparisons

c Unadjusted odds ratio: STORI-30 = 17.810; cells with adjusted standardised residuals ≥+2 *=* Cluster 2 (Moratorium) and Cluster 3 (Growth), cells with adjusted standardised residuals ≤-2 *=* Cluster 2 (Growth) and Cluster 3 (Moratorium).

## Discussion

This study contributes a more comprehensive description of contemporary community rehabilitation unit service users in Australia than has previously been available. Consumers admitted to the CCUs were predominantly males aged in their 30s diagnosed with schizophrenia or related psychotic disorders. Most consumers were born in Australia and had ≤10 years of formal education. Most consumers were referred from community mental health services and had been living with their family before admission. Almost half of the consumers admitted had a current substance use issue, and approximately a quarter had a significant co-morbid physical health issue. Except for current tobacco use, no differences emerged between the study sites on demographic, diagnostic, treatment-related, and consumer-rated variables. However, differences did emerge between the study sites on clinician-rated measures (AUDIT, BPRS, HoNOS, and SFS total scores). The characteristics of the cohort were generally consistent with those defined under the TRR service type in the systematic review by Parker et al. (1). The CA identified three clusters, with differences emerging between the clusters concerning substance use issues, recovery orientation, and levels of symptomatic and functional impairment. This study corroborates the relevance of the consumer characteristics previously defined under the TRR service type.

### Who Uses These Services?

This study provides comprehensive information about consumers admitted to CCUs, including diagnostic, treatment-related, and symptomatic variables. While the primary diagnoses of schizophrenia-spectrum disorders continue to predominate for CCU service users, these occurred less frequently than observed in the modified TRR cohort. This suggests a continuation of the trend towards increased diagnostic heterogeneity of consumers referred to community rehabilitation units identified in the previous systematic review (1). Additionally, the prevalence of substance use comorbidity (44.8%) exceeded that previously identified for TRR type services (21%) (1), and documented in contemporary Australian inpatient rehabilitation services (35–38%) (42, 43). Similarly, the average AUDIT scores in the cohort exceeded the threshold defined for “risky/hazardous drinking”. These findings support the assertion that addressing co-morbid substance use issues, and in particular alcohol use disorders, is an increasingly important consideration for community-based residential rehabilitation services (5, 44). Also, approximately one in four (24.1%) consumers in the cohort were identified as having a significant co-morbid physical health issue; this finding supports recent calls for mental health rehabilitation services to attend the physical health needs of consumers (43, 46).

The impact of under-reporting and non-identification of co-morbidities affecting the cohort must be considered. The prevalence of significant physical health issues was less than that identified in the TRR cohort (36.4%) and in a focused audit of all consumers residing in inpatient and community-based rehabilitation services in Queensland completed in 2016 (46). This audit showed that the metabolic syndrome affected approximately half of these consumers (49.4%). Similarly, issues relating to non-identification and under-reporting of trauma need to be considered given the contrast between the prevalence observed in the cohort (9.7%) and the frequency of childhood trauma reported in the 2010 Australian national survey of psychosis (54.2%) (47). It will be informative to observe if these comorbidities are identified more frequently by the time cohort members are discharged from the CCUs (1).

It is likely that state-based variation in the use of guardianship legislation explains the lower rates of guardianship order use in the current cohort relative to the TRR cohort. This assertion is supported by the disproportionate influence of the data from the South Australia Community Rehabilitation Units on the high frequency of guardianship order use identified in the pooled cohort data (1).

Regarding the symptom-related measures, the average total HoNOS score (i = 10.31) on admission surpassed the threshold for moderate illness severity established by Parabiaghi et al. (48). This average was similar to that observed in Australian samples on admission to inpatient mental health rehabilitation units ($\bar{\text{x}}$ = 9.03-13.49) (42, 43), and on a cross sectional assessment of consumers residing at Queensland CCUs ($\bar{\text{x}}$ = 12.7) (50). Within the clinical assessment battery, both the average total SANS and BPRS scores were also within the ranges approximating Clinical Global Impression-Schizophrenia (CGI-S) scores of ‘mildly ill” (50, 51). The finding that consumers are generally assessed to be mildly-to-moderately-ill based on symptom-related measures is consistent with the conceptualization of CCUs as “non-acute” services (52) and indicates that alarmism about the impact of acute-bed pressures on the function of residential rehabilitation services (5) may not be warranted.

Disability, as assessed by the LSP-16 ($\bar{\text{x}}$ = 12.21), was lower in the cohort than that recently recorded on admission to an Australian non-acute inpatient rehabilitation facility ($\bar{\text{x}}$ = 17.39) (43). This average was also lower than that recorded cross-sectionally for consumers residing at Queensland CCUs in 2014 ($\bar{\text{x}}$ = 17.5) (49). Functional assessment using the ACL indicated that on average, admitted consumers are operating at a level permitting “learning new activity” but with the expectation of needing weekly safety checks and problem-solving assistance (53). With regards to social functioning, the average score on the SFS approximated the 50^th^ percentile of the reference group of unemployed community outpatients with a diagnosis of schizophrenia (33). Overall, the scores on these measures indicate that levels of impairment and disability within the cohort are not extreme relative to other people diagnosed with schizophrenia. This finding is consistent with the CCU service models transitional focus, the expectation of skills development for consumers, and the accommodation structure of self-contained, independent living units (1).

The findings also indicate that consumers are admitted to CCUs at very different stages of their recovery journeys. The stages of recovery most frequently occurring within the cohort were “awareness” (30.5%) and “growth” (30.5%). Andresen et al. describe the “awareness” stage as representing “the person’s dawning realization of the possibility of a more fulfilling life” (p76) with some acknowledgement of personal responsibility for change. In contrast, the “growth” stage reflects an “ongoing dynamic way of living” (p114) with characteristic features including hope, positive future orientation, a sense of personal responsibility and meaning (54). The finding that more than a third of consumers were in the earliest stages of recovery (‘awareness” or “moratorium”) is consistent with staff emphasis on readiness to engage as both barriers and clinical challenges in the delivery of recovery-oriented rehabilitation care at a CCU (5). It may be unrealistic to expect consumers in the “awareness” stage to actively engage in available rehabilitation programs without efforts to build their readiness (55). Building readiness to engage in rehabilitation may involve work around self-awareness, self-efficacy and enhancing motivation by linking interventions with consumers’ goals (55, 56).

The data-driven approach to classification that applied CA to the clinician-rated assessments identified three sub-groups of consumers within the cohort. Differences emerged between the clusters in terms of the levels of symptomatic and functional impairment, as well as substance use issues and stages of recovery. Consumers assigned to Cluster 1 (15%) were characterized by higher levels of co-morbid personality disorder/traits and substance use issues, including specifically alcohol use. Consumers assigned to Cluster 2 (39%) were characterized by higher levels of disability, negative psychotic symptoms and functional impairment; they were also more likely to be in the “moratorium” and less likely to be in the “growth” stage of recovery. Those consumers assigned to Cluster 3 (46%) had lower levels of general psychiatric symptoms and were more likely to be in the “growth” and less likely to be in the “moratorium” stage of recovery. Awareness of the presence of these profiles has implications for service planning and evaluation. Secondary analysis of discharge and follow-up data, when available, based on these clusters, is expected to be informative in terms of their relevance and implications for practice.

Staff working at CCUs have previously identified deficits in their skills to manage comorbid substance use disorders (5), which are issues that often precipitate premature discharge from care (46). The needs of Cluster 1 type consumers could be better met through enhancing staff skills in the management of substance use disorders. Assertive intervention to address alcohol and other substance use issues at the time of admission may facilitate more rapid stabilization for these consumers. Additionally, addressing personality disorder issues concurrently at the time of admission may enhance the stabilization of this sub-group and their engagement with rehabilitation support. One option to achieve this may be external linkage with therapeutic programs available in the community such as Dialectical Behaviour Therapy (57). However, the applicability of these programs to people with comorbid psychotic disorders has not been adequately considered in the literature (58).

The case complexity and higher levels of disability, characterizing consumers assigned to Cluster 2, align well with the CCU model of service (2). The finding that these consumers are more likely to be in the “moratorium” phase and less likely to be in the “growth” phase of recovery has important implications for planning the initial focus of support. Andresen et al. described the “moratorium” stage of recovery as being characterized by “the loss of hope, relinquishment of responsibility for one’s life, loss of a sense of identity and the loss of meaning in life … [contributing to] withdrawal, hopelessness and an apparent lack of motivation” (54) (p53–54). Expecting these consumers to enthusiastically engage with rehabilitation activities at the time admission to the CCU may be both unrealistic and counterproductive. Instead, initial support focused on establishing a sense of hope and expectations of the possibility of recovery is likely to build motivation to engage in rehabilitation activities relevant to their goals (54–56). The higher levels of disability and negative symptoms experienced by Cluster 2 type consumers suggests the relevance of “starting slow” in terms of expectations of engagement. Additionally, these consumers may benefit from a dual focus on skills development as well as mobilization of relevant support to maximize their independence in the community despite the presence of disability.

In contrast consumers assigned to Cluster 3 may be more ready to actively engage with rehabilitation support at the time of the admission. These consumers’ growth orientation aligns with the staff conceptualization of “rehabilitation readiness” (5). However, their higher levels of subjective wellbeing combined with lower levels of disability and symptomatic impairment may mean that their rehabilitation needs may not align as well with the intensity and duration of rehabilitation care available at a CCU as other consumers (e.g. Cluster 2). These consumers may benefit from “starting fast” at the CCU with regards to expectations about therapeutic activity engagement and active work on transition planning from the time of admission.

### What Is the Impact of an Integrated Staffing Model on Admission Patterns?

There were minimal differences identified between the characteristics of consumers admitted to the three study sites. The absence of differences in demographic, diagnostic (except for current tobacco use) and treatment-related variables between sites suggests that similar consumers are being admitted to the units regardless of the staffing configuration. Site-based variation in the clinical assessment battery was observed concerning the total HoNOS, BPRS, SFS and AUDIT measures. However, the pattern was mixed, with only one of these variables (SFS) showed a significant difference between the clinical staffing model and both integrated staffing model sites. Site-based variability may relate to differences in the acuity of referrals at the time of admission or issues with inter-rater reliability, which was not assessed. Overall, the results support the hypothesis that the introduction of an integrated staffing model did not substantially alter the profile of consumers admitted to the CCUs.

### Limitations

These results were derived from a naturalistic observational design, and none of the clinician-rated assessment items were blinded. While orientation was provided to all staff regarding the assessment battery, and training was received by staff in the completion of the routine outcome measures (HoNOS, LSP-16, and MHI-38), inter-rater reliability was not assessed. This may have impacted the reliability of the clinician-rated assessments and the associated comparisons reported between the three study sites. Additionally, most measures in the assessment battery were completed following commencement at the CCU. While the 6-week timeframe applied coincided with the formal assessment period across the sites, the impact of the initial experiences of care cannot be assessed. Qualitative interviews exploring consumers expectations of care completed during this assessment period found positive expectations and favorable comparisons to previous experiences of care and support (20, 1). The effect of the availability of an attractive living environment and the hope for desired “transformation” through receipt of CCU care may have positively impacted the symptomatic and self-report measures.

Several potentially relevant variables were omitted from the available data. Importantly, the assessment battery failed to focus on several factors relating to the planning of rehabilitation care including consumers’ strengths, coping strategies and personal goals (35). Additionally, the perspectives of carers were omitted due to the minimal availability of carer data relative to the size of the consumer cohort. The lack of carer data was driven by the combination of low levels of consumer nomination of carers during the consent process (33.10%), as well as missing data where this consent was provided (18.75%). Carers should be considered a key stakeholder in mental health research (59). Given the high proportion of consumers who had been living with family prior to CCU admission this information would have been informative in understanding the issues contributing to admission to the CCUs.

The generalizability of the results may be limited by the focus on three sites operating within a single health district as well as the approach to statistical grouping within the cohort that was applied. Patterns of referral and admission to community rehabilitation units are likely to be dependent on the mental health and accommodation services array available in the geographic area of interest. The published typology of Australian Community Rehabilitation Units provides a useful reference for considering the generalizability of these findings to other contexts in Australia and internationally (1).

Additionally, the generalizability of CA solutions to wider populations can be limited (8). While CA produces an objective (statistical) grouping, this solution is impacted by the choice of method and the interpretation of the data to identify an optimal solution (7). CA is a hypothesis generating technique. The value of the classification solution to broader decision making about planning the approach to rehabilitation care would be supported by identifying similar clusters within datasets from different sites and TRR service models.

## Conclusions

Consumers admitted to contemporary CCUs are predominantly males, aged in their 30s, diagnosed with schizophrenia spectrum disorders. The characteristics of admitted consumers are similar to those previously defined under the broader TRR cohort. Minimal differences were present in the demographic, diagnostic and treatment-related characteristics of consumers referred across the study sites. While some variation was present in the clinician-rated measures of the clinical assessment battery, there was no clear pattern to suggest that the introduction of an integrated staffing model meaningfully affected the characteristics of consumers admitted for rehabilitation care. The three sub-groups identified through CA were differentiated by the presence of comorbid substance use and personality disorder issues, levels of disability and symptoms, and recovery stage. This classification has potential implications for the planning of rehabilitation care.

## Data Availability Statement

The datasets generated for this study will not be made publicly available. Availability of the datasets associated with this research is limited by ethical approval obtained and would require application for approval to release through the relevant HREC.

## Ethics Statement

The studies involving human participants were reviewed and approved by University of Queensland and Metro South Human Research Ethics Committees (HREC/14/QPAH/62). The patients/participants provided their written informed consent to participate in this study.

## Author Contributions

DH: Provision of expertise and guidance to support the completion of the cluster analysis; review of iterative drafts of the manuscript. DS: Advisory support to SP, including guidance around the study design and process; review of iterative drafts of the manuscript. FD: Content expertise concerning Community Rehabilitation Units; involvement in study design and review of iterative drafts of the manuscript. GM: Review of iterative manuscript drafts; guidance around presentation and analysis of data. HW: Advisory support to SP, including guidance around the initial concept and scope, and methodology; review of iterative manuscript drafts. MH: Advisory support to SP, including guidance around the study design and process, preparation and presentation of data, and interpretation of findings; review of iterative drafts of the manuscript. NK: Contribution to study concept and design; coordination of data collection at one of the study sites; review of iterative drafts of the manuscript. SP: Coordination of the research team; contribution to study concept and design; data collation and analysis; collaborative drafting of the initial manuscript, including identification of key findings and manuscript structure; redrafting manuscript in response to feedback from members of the team. UA: Review of iterative manuscript drafts; guidance around presentation and analysis of data.

## Conflict of Interest

DS, FD, GM, NK, and SP are employees of MSAMHS, a public mental health service in Queensland, Australia that operates the three CCUs study sites that are the subject of investigation. 

The remaining authors declare that the research was conducted in the absence of any commercial or financial relationships that could be construed as a potential conflict of interest.

## Abbreviations

AC-QoL, Adult Carer Quality of Life; ACL, Allen Cognitive Levels; AUDIT, Alcohol Use Disorders Identification Test; ATSI, Persons identifying as being Aboriginal or Torres Strait Islander; BAS, Burden Assessment Scale; BPRS, Brief Psychiatric Rating Scale; CA, Cluster analysis; CR, Carer-rated measure; CL, Clinician-rated measure; CO, Consumer rated/self-report measure; CCU, Community Care Unit; HoNOS, Health of the Nation Outcome Scale; LSP-16, Life Skills Profile-16; MHI-38, Mental Health Inventory; PRPP, Perceive Recall Plan & Perform System of Task Analysis; SANS, Scale for the Assessment of Negative Symptoms; SFS, Social Functioning Scale; SPMI, Severe and Persistent Mental Illness; STORI-30, Stages of Recovery Instrument; TRR, Transitional Residential Rehabilitation.
