# Supplementary material for: A Comprehensive Cohort Description and Statistical Grouping of Community-Based Residential Rehabilitation Service Users in Australia
Source: Front Psychiatry. 2019 Nov 8;10:798. doi: 10.3389/fpsyt.2019.00798 (PMC6857698; doi:10.3389/fpsyt.2019.00798)
Supplement: Supplementary file 1 [file DataSheet_1.pdf]

Demographics of the CCU admission cohort

Staffing model	Clinical	Integrated		Total	Test[e]	p
Site	Site 1 (n=53)	Site 2 (n=52)	Site 3 (n=40)	N=145		
**Demographics**					-	-
Accommodation:					Fisher's Exact Test[f]	.066
- Living with family	56.6%	50.0%	72.5%	58.6%	-	-
- Supported housing	18.9%	5.8%	10.0%	11.7%	-	-
- Private rental	9.4%	15.4%	10.0%	11.7%	-	-
- No fixed address	7.5%	21.2%	2.5%	11.0%	-	-
- Other[a]	7.5%	7.7%	5.0%	6.9%	-	-
Age at admission ($\overline{x}$, years)	31.1 (8.7)	32.1 (8.7)	31.0 (9.8)	31.4 (9.0)	$F_{(2)}$=.214	.808
Male sex	66.0%	78.8%	77.5%	73.8%	$X^2_{(2)}$=2.619	.270
Unemployment[b]	90.6%	82.7%	95.0%	89.0%	$X^2_{(2)}$=3.707	.157
ATSI identification	6.0%	3.8%	10%	6.2%	Fisher's Exact Test[f]	.525
Australian born	86.8%	90.4%	77.5%	85.5%	$X^2_{(2)}$=3.140	.208
Highest education level[c]:					$H_{(2)}$=1.898	.387
- Primary school	5.7%	3.8%	7.5%	5.5%	-	-
- Year 10	41.5%	55.8%	50.0%	49.0%	-	-
- Year 12	34.0%	19.2%	35.0%	29.0%	-	-
- Tertiary[d]	18.9%	19.2%	7.5%	15.9%	-	-

[a] Public housing accounts for 70% of the 'Other' category

[b] Unemployment is exclusive of any form of paid or unpaid vocational activity including volunteering

[c] Treated as a scaled variable based on increasing levels of education, Kruskall-Wallis test applied

[d] Inclusive of any engagement in tertiary education including vocational training regardless of completion

[e] For categorical variables Chi Square test was applied unless the expected count for any cell was <5, in this case Fisher's Exact test was calculated

[f] Unadjusted odds ratio: Accommodation=14.200, ATSI identification=1.500

Primary diagnosis and co-morbidity data for CCU Admission cohort

Staffing model	Clinical	Integrated		Total	Test[b]	p
**Site**	**Site 1 (n=53)**	**Site 2 (n=52)**	**Site 3 (n=40)**	**N=145**		
**Primary diagnosis**					-	-
F20-29.x Schizophrenia spectrum	71.7%	73.1%	90.0%	77.2%	$X^2_{(2)}$=5.143	.076
Specific disorders[a]:					-	-
- F20.x Schizophrenia	47.2%	65.4%	67.5%	59.3%	-	-
- F25.x Schizoaffective disorder	18.9%	5.8%	17.5%	13.8%	-	-
- F29.x Unspecified psychosis	5.7%	1.9%	5.0%	4.1%	-	-
- F31.x Bipolar disorder	15.1%	11.5%	2.5%	10.3%	-	-
- F32-34.x Depressive disorders	5.7%	11.5%	5.0%	7.6%	-	-
- Other disorders	7.4%	3.9%	2.5%	4.9%	-	-
**Secondary diagnoses/issues**					-	-
Current tobacco use	30.2%	73.1%	70.0%	56.6%	$X^2_{(2)}$=23.715	**.000**
Substance use	37.7%	53.8%	42.5%	44.8%	$X^2_{(2)}$=2.875	.237
Physical health issue	22.6%	17.3%	35.0%	24.1%	$X^2_{(2)}$=3.967	.138
Trauma history	9.4%	11.5%	7.5%	9.7%	Fisher's Exact Test[c]	.883
Anxiety disorder	11.3%	9.6%	2.5%	8.3%	Fisher's Exact Test[c]	.290
Developmental disorder	7.5%	9.6%	7.5%	8.3%	Fisher's Exact Test[c]	.932
Personality disorder	5.7%	9.6%	5.0%	6.9%	Fisher's Exact Test[c]	.711
Obsessive-Compulsive Disorder	1.9%	9.6%	2.5%	4.8%	Fisher's Exact Test[c]	.152

[a] Test statistic calculated only for the presence/absence of F20-29.x diagnoses (see above) given the number of diagnostic categories

[b] For categorical variables Chi Square test was applied unless the expected count for any cell was <5, in this case Fisher's Exact test was calculated

[c] Unadjusted odds ratio: Trauma history=0.445, Anxiety disorder=0.256, Developmental disorder=0.266, Personality Disorder=0.890, Obsessive-Compulsive Disorder=3.321

Supplementary Table: Treatment related information for the CCU admission cohort

**Staffing model**	**Clinical**	**Integrated**		**Total**	**Test**	**p**
	**Site 1 (n=53)**	**Site 2 (n=52)**	**Site 3 (n=40)**	**(N=145)**		
**Referral and Legal status**						
Community-based referral	56.6%	63.5%	62.5%	60.7%	$X^2_{(2)}$=.593	.743
Involuntary treatment[b]	52.8%	51.9%	32.5%	46.9%	$X^2_{(2)}$=.4.606	.102
Guardianship order present	5.7%	3.8%	5.0%	4.8%	Fisher's Exact Test[c]	1.000
**Medications prescribed**						
Anti-psychotic medication:						
- CPZ equivalent dose ($\bar{x}$, mg)	436.2 (365.3)	436.4 (284.5)	361.3 (257.7)	415.6 (309.8)	$K_{(2)}$=2.073	.355
- Depot prescribed	45.3%	50.0%	40.0%	45.5%	$X^2_{(2)}$=0.914	.633
- Clozapine prescribed	17.0%	25.0%	37.5%	25.5%	$X^2_{(2)}$=5.061	.080
- Number of antipsychotics	1.36 (0.71)	1.42 (0.696)	1.15 (0.58)	1.32 (0.676)	$K_{(2)}$=4.528	.104
Mood stabiliser:						
- Lithium	20.8%	21.2%	10.0%	17.9%	$X^2_{(2)}$=2.364	.307
- Sodium valproate	9.4%	15.4%	12.5%	12.4%	$X^2_{(2)}$=.855	.652
- Other	7.5%	3.8%	0.0%	4.1%	$X^2_{(2)}$=3.291	.193
Other medication:						
- Antidepressant	41.5%	44.2%	42.5%	42.8%	$X^2_{(2)}$=.081	.960
- Benzodiazepine(s)	13.2%	17.3%	7.5%	13.1%	$X^2_{(2)}$=1.911	.385

[a] Community-based referral compared to combined acute (35.2%) and sub-acute (4.1%) inpatient referral source

[b] Involuntary treatment includes both Involuntary Treatment Orders (43.5%) and Forensic Orders (3.4%)

[c] Unadjusted odds ratio: Guardianship order present =.359

Supplementary Table: Treatment related information for CCU admission cohort (F20-29.x only)

Staffing model	Clinical	Integrated		Total	Test	p
	Site 1 (n=38)	Site 2 (n=38)	Site 3 (n=36)	(N=112)		
**Referral and Legal status**						
Community-based referral[a]	55.3%	65.8%	61.1%	60.7%	$X^2_{(2)}$=.886	.642
Involuntary treatment[b]	57.9%	57.9%	33.3%	50.0%	$X^2_{(2)}$=5.895	.052
Guardianship order present	5.3%	5.3%	5.6%	5.4%	Fisher's Exact Test[c]	1.000
**Medications prescribed**						
Anti-psychotic medication:						
- CPZ equivalent dose ($\overline{x}$, mg)	511.94	511.84	397.73	475.20	$K_{(2)}$=5.445	.355
- Depot prescribed	52.6%	57.9%	44.4%	51.8%	$X^2_{(2)}$=1.356	.508
- Clozapine prescribed	23.7%	34.2%	41.7%	33.0%	$X^2_{(2)}$=2.738	.254
- Number of antipsychotics[d]	1.53(.647)	1.63(.541)	1.25(.500)	1.47(.584)	$K_{(2)}$=8.552	**.014[e]**
Mood stabiliser:						
- Lithium	7.9%	18.4%	8.3%	11.6%	Fisher's Exact Test[c]	.375
- Sodium valproate	7.9%	15.8%	11.1%	11.6%	Fisher's Exact Test[c]	.579
- Other	5.3%	-	-	1.8%	Fisher's Exact Test[c]	.328
Other medication:						
- Antidepressant	34.2%	39.5%	38.9%	37.5%	$X^2_{(2)}$=.268	.874
- Benzodiazepine(s)	10.5%	18.4%	5.6%	11.6%	Fisher's Exact Test[c]	.229

[a] Community-based referral compared to combined acute (34.8%) and sub-acute (4.5%) inpatient referral source

[b] Combined Involuntary Treatment Orders (45.5%) and Forensic Orders (4.5%), compared to voluntary status

[c] Unadjusted odds ratio: Guardianship order present = .226, Lithium prescribed = 2.338, Sodium valproate prescribed = 1.157, Other mood stabiliser prescribed = 2.630, Benzodiazepines prescribed = 2.855.

[d] Mean score, standard deviations is provided in brackets

[e] Post-hoc tests with Bonferroni correction for multiple tests identified statistically significant pairwise comparison between Sites 2 and 3 only (p=.013)

Supplementary Table: Clinical assessment battery for the CCU admission cohort, mean scores and standard deviation

Staffing model		Clinical		Integrated				Total		
		Site 1		Site 2		Site 3				
	n	$\bar{x}$(SD)	n	$\bar{x}$(SD)	n	$\bar{x}$(SD)	N[a]	$\bar{x}$(SD)	Test	p
**Functioning and disability**										
HoNOS (Total)	53	8.98(6.125)	51	9.75(4.707)	40	12.80(6.638)	144	10.31(5.992)	$K_{(2)}$=9.444	**.009**[b]
- Behaviour		1.13(1.699)		1.06(1.475)		1.20(1.682)		1.13(1.608)	$K_{(2)}$=.169	.919
- Impairment		1.38(1.431)		1.49(1.317)		2.48(1.633)		1.72(1.517)	$K_{(2)}$=13.293	**.001**[c]
- Symptoms		2.74(2.159)		3.65(2.331)		4.55(2.501)		3.56(2.414)	$K_{(2)}$=12.303	**.002**[d]
- Social		3.74(2.995)		3.55(2.766)		4.58(3.161)		3.90(2.974)	$K_{(2)}$=2.740	.254
LSP-16 (Total)	50	10.78(5.643)	51	12.53(6.166)	39	13.62(5.775)	140	12.21(5.945)	$K_{(2)}$=5.262	.072
- Withdrawal		2.58(1.864)		2.92(1.864)		3.26(1.860)		2.89(1.869)	$K_{(2)}$=4.042	.132
- Self-care		3.38(2.118)		4.53(2.411)		5.38(2.208)		4.36(2.378)	$K_{(2)}$=16.799	**.000**[b]
- Compliance		2.14(1.539)		2.10(1.652)		1.77(1.512)		2.02(1.571)	$K_{(2)}$=1.036	.596
- Anti-social		1.48(1.515)		1.43(1.723)		1.46(1.620)		1.46(1.611)	$K_{(2)}$=.212	.899
Allen Cognitive Level	51	5.03 (.405)	48	5.16(.4261)	40	5.01(337)	139	5.07(.398)	$K_{(2)}$=5.345	.069
Social Functioning Scale	51	107.05(7.814)	50	102.95(7.996)	39	100.84(7.784)	140	103.85(8.224)	$K_{(2)}$=13.362	**.001**[f]
**Symptomatic measures***										
BPRS-18 (Total)	51	37.47(8.889)	46	36.67(9.778)	36	42.81(9.730)	133	38.64(9.707)	$K_{(2)}$=8.162	**.017**[g]
- Resistance		6.08(2.606)		5.89(1.816)		5.92(2.285)		5.97(2.256)	$K_{(2)}$=.290	.865
- Positive symptoms		10.51(5.108)		10.33(4.634)		12.25(5.369)		10.92(5.054)	$K_{(2)}$=3.337	.189
- Negative symptoms		6.76(3.664)		6.02(3.363)		7.69(3.060)		6.76(3.442)	$K_{(2)}$=6.790	**.034**[h]
- Psychological discomfort		13.00(4.152)		13.09(5.001)		15.50(5.364)		13.71(4.888)	$F_{(2,130)}$=3.449	**.035**[i]
SANS (Total)	51	43.53(18.884)	49	49.53(16.686)	36	50.61(18.243)	136	47.57(18.094)	$F_{(2,133)}$=2.102	.126
- Affective flattening		14.65(8.756)		14.90(8.295)		14.94(8.349)		14.77(8.442)	$K_{(2)}$=.122	.941
- Alogia		3.29(4.125)		4.86(4.168)		5.58(4.129)		4.53(4.267)	$K_{(2)}$=10.231	**.006**[j]
- Avolition/apathy		8.71(4.494)		10.27(2.782)		10.64(3.331)		9.78(3.699)	$K_{(2)}$=8.643	**.013**[k]

- Anhedonia/asociality		13.41(5.193)		15.08(3.834)		14.69(4.125)		14.29(4.553)	$F_{(2,133)}$=1.892	.155
- Attention		3.47(3.349)		4.43(3.482)		4.75(2.802)		4.17(.374)	$K_{(2)}$=4,564	.102
**Substance use (alcohol)**										
AUDIT	48	4.90(7.856)	50	10.38(10.111)	35	6.80(6.957)	133	7.46(8.839)	$K_{(2)}$=12.809	**.002**[l]

[a] Available sample size varies based on missing data: HoNOS (.9%), LSP-16 (3.5%), Allens Cognitive Level (4.1%), SFS (3.4%), BPRS-18 (8.3%), SANS (6.2%) and AUDIT (8.3%)

[b] Post-hoc tests with Bonferroni correction for multiple tests identified statistically significant pairwise comparison between Sites 1 and 3 only (p=.006)

[c] Post-hoc tests with Bonferroni correction for multiple tests identified statistically significant pairwise comparison between Site 3 and Sites 1&2 (p=.002, p.011 respectively)

[d] Post-hoc tests with Bonferroni correction for multiple tests identified statistically significant pairwise comparison between Sites 1 and 3 only (p=.002)

[e] Post-hoc tests with Bonferroni correction for multiple tests identified statistically significant pairwise comparison between Site 1 and Sites 2&3 (p=.033, p=.017 respectively)

[f] Post-hoc tests with Bonferroni correction for multiple tests identified statistically significant pairwise comparison between Site 1 and Sites 2&3 (p=.034, p=.001 respectively)

[g] Post-hoc tests with Bonferroni correction for multiple tests identified statistically significant pairwise comparison between Sites 2 and 3 only (p=.023)

[h] Post-hoc tests with Bonferroni correction for multiple tests identified statistically significant pairwise comparison between Sites 2 and 3 only (p=.029)

[i] Post-hoc tests with Bonferroni correction for multiple tests identified no statistically significant pairwise comparisons

[j] Post-hoc tests with Bonferroni correction for multiple tests identified statistically significant pairwise comparison between Sites 1 and 2&3 (p=.043, p=.010 respectively)

[k] Post-hoc tests with Bonferroni correction for multiple tests identified statistically significant pairwise comparison between Sites 1 and 3 only (p=.022)

[l] Post-hoc tests with Bonferroni correction for multiple tests identified statistically significant pairwise comparison between Sites 1 and 2 only (p=.001)

Supplementary Table: Clinical assessment battery for the CCU admission cohort (F20-29.x only)

**Staffing model**		**Clinical**		**Integrated**				**Total**		
		**Site 1**		**Site 2**		**Site 3**				
	**n**	**$\bar{x}$(SD)**	**n**	**$\bar{x}$(SD)**	**n**	**$\bar{x}$(SD)**	**N**[a]	**$\bar{x}$(SD)**	**Test**	**p**
**Functioning and disability**										
HoNOS (Total)	38	7.53(4.864)	37	9.27(4.593)	36	11.97(6.194)	111	9.55(5.516)	$K_{(2)}$=10.912	**.004**[b]
- Behaviour		.66(1.097)		.86(1.084)		1.03(1.424)		.85(1.208)	$K_{(2)}$=1.768	.413
- Impairment		1.18(1.333)		1.41(1.189)		2.42(1.713)		1.66(1.511)	$K_{(2)}$=11.401	**.003**[c]
- Symptoms		2.66(2.233)		3.57(2.534)		4.44(2.568)		3.54(2.533)	$K_{(2)}$=9.376	**.009**[d]
- Social		3.03(2.433)		3.43(2.774)		4.08(2.812)		3.50(2.686)	$K_{(2)}$=2.798	.247
LSP-16 (Total)	38	22.97(16.714)	38	18.08(12.052)	36	16.43(6.464)	108	19.18(12.698)	$K_{(2)}$=2.508	.285
- Withdrawal		6.61(4.435)		5.89(3.821)		5.37(2.669)		5.96(3.724)	$K_{(2)}$=.883	.643
- Self-care		6.78(5.772)		5.76(3.655)		6.57(3.319)		6.36(4.370)	$K_{(2)}$=1.829	.401
- Compliance		3.89(4.328)		2.78(2.200)		2.26(2.331)		2.98(3.153)	$K_{(2)}$=3.831	.147
- Anti-social		5.69(6.769)		3.65(6.061)		2.23(3.163)		3.87(5.705)	$K_{(2)}$=6.326	**.042**[e]
Allen Cognitive Level	37	5.03(.409)	36	5.100(.4071)	36	4.99(.351)	109	5.039(.389)	$F_{(2,106)}$=.755	.473
Social functioning scale	37	106.47(8.248)	36	103.38(8.320)	35	101.54(7.641)	108	103.84(8.263)	$F_{(2,105)}$=3.432	**.036**[f]
**Symptomatic measures***										
BPRS-18 (Total)	36	39.31(9.099)	34	38.47(9.589)	33	43.09(10.110)	103	40.24(9.706)	$K_{(2)}$=3.175	.204
- Resistance		6.33(2.878)		6.00(1.792)		5.67(2.010)		6.01(2.286)	$K_{(2)}$=1.234	.540
- Positive symptoms		11.94(5.231)		11.26(4.654)		12.79(3.442)		11.99(5.054)	$K_{(2)}$=1.266	.531
- Negative symptoms		6.92(3.442)		6.74(3.587)		7.79(3.180)		7.14(3.407)	$K_{(2)}$=2.502	.286
- Psychological discomfort		13.06(4.362)		13.00(4.905)		15.36(5.550)		13.78(5.017)	$F_{(2,100)}$=2.501	.087
SANS (Total)	36	43.89(14.790)	35	54.91(15.334)	33	51.09(18.603)	104	52.62(16.305)	$F_{(2,104)}$=1.243	.293
- Affective flattening		17.22(7.620)		16.74(8.154)		15.30(8.509)		16.45(8.052)	$K_{(2)}$=.990	.610
- Alogia		3.36(3.773)		6.11(4.185)		5.70(4.104)		5.03(4.168)	$K_{(2)}$=10.708	**.005**[g]
- Avolition/apathy		9.75(3.894)		10.63(2.568)		10.55(3.465)		10.30(3.350)	$F_{(2,104)}$=1.226	.298

- Anhedonia/asociality		15.08(4.423)		16.00(3.804)		14.61(4.153)		15.24(4.137)	$K_{(2)}$=1.961	.375
- Attention		3.47(3.229)		5.43(3.517)		4.94(2.828)		4.60(3.290)	$K_{(2)}$=5.585	.061
**Substance use**										
AUDIT	36	5.78(8.839)	37	9.89(9.776)	32	6.69(6.860)	105	7.60(8.752)	$K_{(2)}$=6.700	**.029**[h]

[a] Available sample size varies based on missing data: HoNOS (.9%), LSP-16 (3.6%), Allens Cognitive Level (2.7%), BPRS-18 (8.0%), SANS (7.1%) and AUDIT (6.7%)
[b] Post-hoc tests with Bonferroni correction for multiple tests identified statistically significant pairwise comparison between Sites 1 and 3 only (p=.003)
[c] Post-hoc tests with Bonferroni correction for multiple tests identified statistically significant pairwise comparison between Sites 1 and 3 only (p=.003)
[d] Post-hoc tests with Bonferroni correction for multiple tests identified statistically significant pairwise comparison between Sites 1 and 3 only (p=.007)
[e] Post-hoc tests with Bonferroni correction for multiple tests identified statistically significant pairwise comparison between Sites 1 and 3 only (p=.047)
[f] Post-hoc tests with Bonferroni correction for multiple tests identified statistically significant pairwise comparison between Sites 3 and 1&2 (p=.033, p=.028 respectively)
[g] Post-hoc tests with Bonferroni correction for multiple tests identified statistically significant pairwise comparison between Sites 1 and 2&3 (p=.008, p=.029 respectively)
[h] Post-hoc tests with Bonferroni correction for multiple tests identified statistically significant pairwise comparison between Sites 1 and 3 only (p=.029)

Supplementary Table Consumer rated assessments for the CCU admission cohort

Staffing model	Clinical		Integrated				Total			
	Site 1		Site 2		Site 3					
	n	$\bar{x}$(SD)	n	$\bar{x}$(SD)	n	$\bar{x}$(SD)	N[a]	$\bar{x}$(SD)	Test	p
**Mental Health Inventory (Total)**	52	57.52(17.076)	52	53.17(21.787)	40	56.40(21.358)	144	55.64(20.034)	$K_{(2)}$=1.899	.387
Psychological wellbeing		47.50(21.775)		41.92(25.469)		47.08(19.737)		45.37(22.660)	$K_{(2)}$=1.488	.475
Psychological distress		34.04(21.718)		38.90(24.579)		32.60(24.674)		35.40(23.602)	$K_{(2)}$=1.385	.500
**STORI-30**	45	-	47	-	40	-	132	-	Fisher's exact[b]	.318
Moratorium	7	15.6%	5	10.6%	2	5.0%	14	10.6%		
Awareness	17	37.8%	10	21.3%	18	45.0%	45	34.1%		
Preparation	3	6.7%	3	6.4%	3	7.5%	9	6.8%		
Rebuilding	7	15.6%	13	27.7%	6	15.0%	26	19.7%		
Growth	11	24.4%	16	34.0%	11	27.5%	38	28.8%		

[a] Available sample size varies based on missing data: MHI (.6%), STORI-30 (9.0%)
[b] Unadjusted odds ratio: STORI-30 = 9.228

Consumer rated assessments for the CCU admission cohort (F20-29.x only)

**Staffing model**	**Clinical**		**Integrated**				**Total**			
	**Site 1**		**Site 2**		**Site 3**					
	**n**	**$\bar{x}$(SD)**	**n**	**$\bar{x}$(SD)**	**n**	**$\bar{x}$(SD)**	**N[a]**	**$\bar{x}$(SD)**	**Test**	**p**
**Mental Health Inventory (Total)**	37	56.95(18.571)	38	55.61(21.104)	36	57.44(21.771)	111	56.65(20.350)	$K_{(2)}$=.737	.692
Psychological wellbeing		47.42(23.197)		45.29(24.851)		48.61(18.958)		47.08(22.363)	$F_{(2,108)}$=.208	.813
Psychological distress		35.97(22.060)		36.50(24.837)		31.03(24.997)		34.55(23.91)	$K_{(2)}$=1.713	.425
**STORI-30**	32		34		36		102		Fisher's Exact[b]	.573
Moratorium	6	18.8%	3	8.8%	2	5.6%	11	10.8%		
Awareness	11	34.4%	8	23.5%	15	41.7%	34	33.3%		
Preparation	2	6.3%	2	5.9%	2	5.6%	6	5.9%		
Rebuilding	6	18.8%	10	29.4%	6	16.7%	22	21.6%		
Growth	7	21.9%	11	32.4%	11	30.6%	29	28.4%		

[a] Available sample size varies based on missing data: MHI (.9%), STORI-30 (8.9%)
[b] Unadjusted odds ratio: STORI-30 = 6.741

Supplementary Table: Consumer rated assessments for the CCU admission cohort (F20-29.x only)

Staffing model	Clinical		Integrated				Total			
	Site 1		Site 2		Site 3					
	n	$\bar{x}$(SD)	n	$\bar{x}$(SD)	n	$\bar{x}$(SD)	N[a]	$\bar{x}$(SD)	Test	p
**Mental Health Inventory (Total)**	37	56.95(18.571)	38	55.61(21.104)	36	57.44(21.771)	111	56.65(20.350)	$K_{(2)}$=.737	.692
Psychological wellbeing		47.42(23.197)		45.29(24.851)		48.61(18.958)		47.08(22.363)	$F_{(2,108)}$=.208	.813
Psychological distress		35.97(22.060)		36.50(24.837)		31.03(24.997)		34.55(23.91)	$K_{(2)}$=1.713	.425
**STORI-30**	32		34		36		102		Fisher's Exact[b]	.573
Moratorium	6	18.8%	3	8.8%	2	5.6%	11	10.8%		
Awareness	11	34.4%	8	23.5%	15	41.7%	34	33.3%		
Preparation	2	6.3%	2	5.9%	2	5.6%	6	5.9%		
Rebuilding	6	18.8%	10	29.4%	6	16.7%	22	21.6%		
Growth	7	21.9%	11	32.4%	11	30.6%	29	28.4%		

[a] Available sample size varies based on missing data: MHI (.9%), STORI-30 (8.9%)
[b] Unadjusted odds ratio: STORI-30 = 6.741

Supplementary Table: Comparisons between the cohort and the modified TRR cohort from Parker et al (2019)

		TRR cohort (n)	Admission cohort (n)	Chi-square	p
**Sex**	Male	733	107	.11	.744
	Female	278	38	-	-
**Australian born**	Yes	559	124	.03	.863
	No	99	21	-	-
**ATSI identification**	Yes	90	9	1.46	.228
	No	882	136	-	-
**Unemployment**	Yes	169	129	18.3	**.000**
	No	72	16	-	-
**Community-based referral**	Yes	375	88	1.2	.274
	No	298	57	-	-
**Involuntary treatment**	Yes	534	68	.25	.6134
	No	553	77	-	-
**Guardianship order**	Yes	321	7	71.6	**.000**
	No	452	138	-	-
**Diagnosis F20-29.x**	Yes	970	112	8.05	**.005**
	No	156	33	-	-
**Substance use issue**	Yes	184	65	40.5	**.000**
	No	712	80	-	-
**-Developmental disorders**	Yes	13	12	5.9	**.015**
	No	380	133	-	-
**Personality disorder**	Yes	19	10	.88	.347
	No	374	135	-	-
**Physical health issues**	Yes	396	35	8.5	**.004**
	No	691	110	-	-
**Age (mean)**	-	35.5	31.4	-	-
**LSP Total (mean)**	-	17.5	19.1	-	-
**HoNOS Total (mean)**	-	15.3	10.3	-	-
**CPZ Dose equivalence (mean)**	-	612.5	415.6	-	-

Supplementary table: Demographics by cluster

Site	Cluster 1 (n=17)	Cluster 2 (n=43)	Cluster 3 (n=51)	TOTAL (N=111)		
**Demographics**					-	-
Age in years at admission ($\bar{x}$, SD)	31.35(7.441)	32.98(10.809)	29.82(8.294)	31.28(9.276)	$F_{(2,108)}=1.357$	.262
Male sex	76.5%	79.1%	70.6%	74.8%	Fisher's Exact Test	.669
Australian born	88.2%	90.7%	86.3%	88.3%	Fisher's Exact Test	.857
ATSI identification	17.6%	4.9%	6.3%	7.2%	Fisher's Exact Test	.178
Unemployment[a]	82.4%	97.7%	90.2%	91.9%	Fisher's Exact Test	.090
**Accommodation (most recent)**					Fisher's Exact Test	.133
Living with family	58.8%	55.8%	62.7%	59.5%	-	-
Supported housing	5.9%	20.9%	5.9%	11.7%	-	-
Private rental	23.5%	7.0%	9.8%	10.8%	-	-
No fixed address	5.9%	7.0%	17.6%	11.7%	-	-
Other[b]	5.9%	9.3%	3.9%	6.3%	-	-
**Highest education level[c]**					$H_{(2)}=3.538$	.171
Primary school	5.9%	4.7%	7.8%	6.3%	-	-
Year 10	52.9%	55.8%	35.3%	45.9%	-	-
Year 12	35.3%	25.6%	35.3%	31.5%	-	-
Tertiary[d]	5.9%	14.0%	21.6%	16.2%	-	-

[a] Unemployment is exclusive of any form of paid or unpaid vocational activity including volunteering
[b] Public housing accounts for 70% of the 'Other' category
[c] Treated as a scaled variable based on increasing levels of education, Kruskal-Wallis test applied
[d] Inclusive of any engagement in tertiary education including vocational training regardless of completion

Supplementary Table: Diagnosis by cluster

	Cluster 1 (n=17)	Cluster 2 (n=43)	Cluster 3 (n=51)	TOTAL (N=111)	Test[b]	p
**Primary diagnosis[a]**					-	-
F20-29.x Schizophrenia spectrum	82.4%	88.4%	72.5%	80.2%	Fisher's Exact Test[c]	.156
Specific disorders[a]:					-	-
- F20.x Schizophrenia	64.7%	60.5%	64.7%	63.1%	-	-
- F25.x Schizoaffective disorder	17.6%	20.9%	3.9%	12.6%	-	-
- F29.x Unspecified psychosis	-	7.0%	3.9%	4.5%	-	-
- F31.x Bipolar disorder	11.8%	2.3%	13.8%	9.0%	-	-
- F32-34.x Depressive disorders	5.9%	7.0%	5.9%	6.3%	-	-
- Other disorders	-	2.3%	3.9%	2.7%	-	-
**Secondary diagnoses/issues**					-	-
Current tobacco use	70.6%	65.1%	47.1%	57.7%	$X^2_{(2)}=4.491$	.106
Substance use	94.1%	32.6%	35.3%	43.2%	$X^2_{(2)}=21.240$	**.000**[d]
Physical health issue	11.8%	27.9%	17.6%	20.7%	Fisher's Exact Test[c]	.353
Trauma history	5.9%	2.3%	11.8%	7.2%	Fisher's Exact Test[c]	.207
Anxiety disorder	5.9%	4.7%	15.7%	9.9%	Fisher's Exact Test[c]	.191
Developmental disorder	5.9%	4.7%	13.3%	8.1%	Fisher's Exact Test[c]	.456
Personality disorder	23.5%	4.7%	3.9%	7.2%	Fisher's Exact Test[c]	**.042**[e]
Obsessive-Compulsive Disorder	-	9.3%	3.9%	5.4%	Fisher's Exact Test[c]	.447

[a] Test statistic calculated only for the presence/absence of F20-29.x diagnoses (see above) given the number of diagnostic categories
[b] For categorical variables, the Chi Square test was applied unless the expected count for any cell was <5, in this case, Fisher's Exact test was calculated
[c] Unadjusted odds ratio: F20-29.x Schizophrenia spectrum=3.628, Substance use=22.60, Physical health issue=2.239; Trauma history=2.943; Trauma history=3.099; Developmental disorder=1.513; Personality disorder=6.082; Obsessive-Compulsive Disorder=1.787
[d] Cells with adjusted standardised residuals ≥+2= Cluster 1 (Substance use issue – Yes)
[e] Cells with adjusted standardised residuals ≥+2= Cluster 1 (Personality Disorder – Yes)

Supplementary Table: Treatment variables by cluster

	Cluster 1 (n=17)	Cluster 2 (n=43)	Cluster 3 (n=51)	TOTAL (N=111)	Test[b]	p
**Referral and Legal status**						
Community-based referral	52.9%	69.8%	62.7%	64.0%	$X^2_{(2)}$=1.557	.459
Involuntary treatment[b]	41.2%	41.9%	41.2%	41.4%	$X^2_{(2)}$=.005	.997
Guardianship order present	-	2.3%	7.8%	4.5%	Fisher's Exact Test[c]	.459
**Medications prescribed**						
Anti-psychotic medication:						
- CPZ equivalence, mg ($\overline{x}$, SD)	522.2(359.7)	391.5(212.1)	382.6(322.7)	407.41(292.9)	$K_{(2)}$=2.373	.305
- Depot prescribed	52.9%	46.5%	43.1%	45.9%	$X^2_{(2)}$=.502	.778
- Clozapine prescribed	29.4%	16.3%	27.5%	23.4%	Fisher's Exact Test[c]	.336
- Number of antipsychotics	1.41(.618)	1.28(.630)	1.31(.678)	1.32(.676)	$K_{(2)}$=.756	.685
Mood stabiliser:						
- Lithium	23.5%	16.3%	5.9%	14.4%	Fisher's Exact Test[c]	.506
- Sodium valproate	11.8%	16.3%	5.9%	10.8%	Fisher's Exact Test[c]	.291
- Other	-	2.3%	7.8%	4.5%	Fisher's Exact Test[c]	.459
Other medication:						
- Antidepressant	29.4%	51.2%	37.3%	41.4%	$X^2_{(2)}$=3.057	.217
- Benzodiazepine(s)	17.6%	16.3%	5.9%	11.7%	Fisher's Exact Test[c]	.174

[a] Community-based referral compared to combined acute and sub-acute inpatient referral source

[b] Involuntary treatment includes both Involuntary Treatment Orders and Forensic Order

[c] Unadjusted odds ratio: Guardianship order present =1.758, Clozapine prescribed=2.139; Lithium prescribed=1.545; Sodium valproate prescribed=2.718; Other mood stabiliser prescribed=1.758; Benzodiazepine(s)=3.419

Supplementary Table: Clinical assessment battery – mean score and (standard deviation) by cluster

	Cluster 1 (n=17)		Cluster 2 (n=43)		Cluster 3 (n=51)		Total (N=111)			
	$\bar{x}$	(SD)	$\bar{x}$	(SD)	$\bar{x}$	(SD)	$\bar{x}$	(SD)	Test	p
**Functioning and disability**										
HoNOS (Total)	9.00	5.534	13.98	5.755	6.94	3.906	9.98	5.891	$K_{(2)}=35.674$	**.000**[a]
- Behaviour	1.88	1.409	1.21	1.567	.51	1.255	.99	1.480	$K_{(2)}=21.306$	**.000**[b]
- Impairment	1.41	1.734	2.40	1.482	1.10	1.237	1.65	1.529	$K_{(2)}=18.997$	**.000**[c]
- Symptoms	3.65	2.548	4.84	2.468	2.41	1.878	3.54	2.475	$K_{(2)}=21.968$	**.000**[d]
- Social	2.06	2.384	5.53	3.150	2.92	2.162	3.80	2.957	$K_{(2)}=24.865$	**.000**[e]
LSP-16 (Total)	8.65	5.219	16.74	5.416	9.33	4.339	12.21	5.945	$K_{(2)}=40.508$	**.000**[e]
- Withdrawal	1.53	1.218	4.37	1.865	2.18	1.352	2.92	1.882	$K_{(2)}=41.562$	**.000**[e]
- Self-care	3.59	2.717	5.81	2.119	3.31	1.715	4.40	2.389	$K_{(2)}=30.522$	**.000**[f]
- Compliance	1.47	1.625	2.49	1.549	1.65	1.494	2.06	1.603	$K_{(2)}=8.697$	**.013**[g]
- Anti-social	1.18	1.237	1.84	1.717	1.18	1.545	1.45	1.610	$K_{(2)}=4.711$	.095
Allen Cognitive Level	4.95	.445	5.00	.389	5.13	.407	5.054	.409	$K_{(2)}=3.714$	.156
Social Functioning Scale	104.57	8.183	98.02	7.138	107.82	6.95	103.53	8.469	$K_{(2)}=34.695$	**.000**[h]
**Symptomatic measures**										
BPRS-18 (Total)	39.24	8.066	44.33	8.225	32.41	6.885	38.07	9.338	$K_{(2)}=38.473$	**.000**[i]
- Resistance	5.76	1.954	6.67	2.476	5.04	1.536	5.78	2.129	$K_{(2)}=13.685$	**.001**[j]
- Positive symptoms	11.29	4.089	12.12	5.399	9.08	3.893	10.59	4.743	$K_{(2)}=9.232$	**.010**[k]
- Negative symptoms	6.24	2.728	8.63	3.599	5.22	2.648	6.69	3.424	$K_{(2)}=22.763$	**.000**[l]
- Psychological discomfort	14.94	4.220	15.30	5.040	11.98	4.474	13.72	4.899	$F_{(2,108)}=6.595$	**.002**[m]
SANS (Total)	45.82	11.706	58.63	14.635	39.41	16.755	47.84	17.561	$F_{(2,108)}=18.616$	**.000**[n]
- Affective flattening	14.18	7.427	18.67	7.177	11.63	7.997	14.75	8.207	$K_{(2)}=16.675$	**.000**[l]
- Alogia	3.65	3.040	6.53	4.677	3.24	3.479	4.58	4.203	$K_{(2)}=14.393$	**.001**[j]
- Avolition/apathy	9.76	2.818	11.88	2.312	8.47	3.797	9.99	3.497	$K_{(2)}=25.374$	**.000**[o]

- Anhedonia/asociality	13.88	3.295	16.79	3.433	12.61	4.618	14.42	4.420	$K_{(2)}$=21.703	**.000**[p]
- Attention	4.35	3.081	4.74	3.430	3.47	2.976	4.10	3.202	$K_{(2)}$=3.383	.184
**Substance use (alcohol)**										
AUDIT	23.53	6.983	5.67	6.171	3.88	4.48	7.59	8.823	$K_{(2)}$=42.393	**.000**[q]

a Post-hoc tests with Bonferroni correction for multiple tests identified statistically significant pairwise comparison between Cluster 2 and 1&3 (p=.013 and p.000 respectively)

b Post-hoc tests with Bonferroni correction for multiple tests identified statistically significant pairwise comparison between Cluster 3 and 1&2 (p=.008 and p.000 respectively)

c Post-hoc tests with Bonferroni correction for multiple tests identified statistically significant pairwise comparison between Cluster 2 and 1&3 (p=.024 and p.000 respectively)

d Post-hoc tests with Bonferroni correction for multiple tests identified statistically significant pairwise comparison between Cluster 2 and 3 only (p=.000)

e Post-hoc tests with Bonferroni correction for multiple tests identified statistically significant pairwise comparison between Cluster 2 and 1&3 (p=.000 and p.000 respectively)

f Post-hoc tests with Bonferroni correction for multiple tests identified statistically significant pairwise comparison between Cluster 2 and 1&3 (p=.001 and p.000 respectively)

g Post-hoc tests with Bonferroni correction for multiple tests identified statistically significant pairwise comparison between Cluster 2 and 3 only (p=.029)

h Post-hoc tests with Bonferroni correction for multiple tests identified statistically significant pairwise comparison between Cluster 2 and 1&3 (p=.016 and p.000 respectively)

i Post-hoc tests with Bonferroni correction for multiple tests identified statistically significant pairwise comparison between Cluster 3 and 1&2 (p=.016 and p.000 respectively)

j Post-hoc tests with Bonferroni correction for multiple tests identified statistically significant pairwise comparison between Cluster 2 and 3 only (p=.001)

k Post-hoc tests with Bonferroni correction for multiple tests identified statistically significant pairwise comparison between Cluster 2 and 3 only (p=.013)

l Post-hoc tests with Bonferroni correction for multiple tests identified statistically significant pairwise comparison between Cluster 2 and 3 only (p=.000)

m Post-hoc tests with Bonferroni correction for multiple tests identified statistically significant pairwise comparison between Cluster 2 and 3 only (p=.003)

n Post-hoc tests with Bonferroni correction for multiple tests identified statistically significant pairwise comparison between Cluster 1 and 2 (p=.013), and Cluster 2 and 3 (p=.000)

0 Post-hoc tests with Bonferroni correction for multiple tests identified statistically significant pairwise comparison between Cluster 2 and 1&3 (p=.025 and p.000 respectively)

p Post-hoc tests with Bonferroni correction for multiple tests identified statistically significant pairwise comparison between Cluster 2 and 1&3 (p=.034 and p.000 respectively)

q Post-hoc tests with Bonferroni correction for multiple tests identified statistically significant pairwise comparison between Cluster 1 and 2&3 (p=.000 and p.000 respectively)

Supplementary Table: Consumer-rated measures by Cluster

	Cluster 1		Cluster 2		Cluster 3					
	**n**	**$\bar{x}$(SD)**	**n**	**$\bar{x}$(SD)**	**n**	**$\bar{x}$(SD)**	**N[a]**	**$\bar{x}$(SD)**	**Test**	**p**
**Mental Health Inventory (Total)**	17	49.88(22.209)	43	51.84(19.848)	51	63.00(17.034)	111	56.67(19.720)	$K_{(2)}$=10.445	**.005**[a]
Psychological wellbeing		38.53(23.492)		40.00(22.018)		53.31(21.575)		45.89(22.901)	$K_{(2)}$=11.118	**.004[b]**
Psychological distress		43.41(24.308)		39.26(22.065)		27.12(22.230)		34.32(23.298)	$K_{(2)}$=7.836	**.020**[c]
**STORI-30**	16	-	42	-	47	-	105	-	Fisher's exact[b]	**.015**[d]
Moratorium	3	18.8%	9	21.4%	2	4.3%	14	13.3%		
Awareness	7	43.8%	15	35.7%	10	21.3%	32	30.5%		
Preparation	1	6.3%	2	4.8%	6	12.8%	9	8.6%		
Rebuilding	2	12.5%	9	21.4%	7	14.9%	18	17.1%		
Growth	3	18.8%	7	16.7%	22	46.8%	32	30.5%		

a Post-hoc tests with Bonferroni correction for multiple tests identified statistically significant pairwise comparison between Cluster 3 and 1&2 (p=.030 and p.021 respectively)

b Post-hoc tests with Bonferroni correction for multiple tests identified statistically significant pairwise comparison between Cluster 3 and 1&2 (p=.042 and p.010 respectively)

c Post-hoc tests with Bonferroni correction for multiple tests identified no statistically significant pairwise comparisons

d Unadjusted odds ratio: STORI-30 = 17.810; cells with adjusted standardised residuals ≥+2= Cluster 2 (Moratorium) and Cluster 3 (Growth), cells with adjusted standardised residuals ≤-2= Cluster 2 (Growth) and Cluster 3 (Moratorium).

Supplementary Information: Cluster solution (Figure 1)

Z-scores for clinical assessment battery items

	N				Mean				Std. Error			
	1	2	3	Total	1	2	3	Total	1	2	3	Total
Zscore: LSP_TOTAL_recode	17	43	51	111	-0.60	0.76	-0.48	-0.02	0.21	0.14	0.10	0.10
Zscore: HoNOS Total score (re-coded)	17	43	51	111	-0.22	0.61	-0.56	-0.06	0.22	0.15	0.09	0.09
Zscore: BPRS Total score	17	43	51	111	0.06	0.59	-0.64	-0.06	0.20	0.13	0.10	0.09
Zscore: SANS Total	17	43	51	111	-0.10	0.61	-0.45	0.02	0.16	0.12	0.13	0.09
Zscore: AUDIT Total score	17	43	51	111	1.82	-0.20	-0.40	0.01	0.19	0.11	0.07	0.09
Zscore: SFS - Average Scaled	17	43	51	111	0.09	-0.71	0.48	-0.04	0.24	0.13	0.12	0.10
Zscore: Allen's Cognitive Level	17	43	51	111	-0.29	-0.19	0.17	-0.04	0.27	0.15	0.14	0.10

Conversion so that positive scores equate to less impairment for all variables

	N				Mean				Std. Error			
	1	2	3	Total	1	2	3	Total	1	2	3	Total
Zscore: LSP_TOTAL_recode	17	43	51	111	0.60	-0.76	0.48	0.02	0.21	0.14	0.10	0.10
Zscore: HoNOS Total score (re-coded)	17	43	51	111	0.22	-0.61	0.56	0.06	0.22	0.15	0.09	0.09
Zscore: BPRS Total score	17	43	51	111	-0.06	-0.59	0.64	0.06	0.20	0.13	0.10	0.09
Zscore: SANS Total	17	43	51	111	0.10	-0.61	0.45	-0.02	0.16	0.12	0.13	0.09
Zscore: AUDIT Total score	17	43	51	111	-1.82	0.20	0.40	-0.01	0.19	0.11	0.07	0.09
Zscore: SFS - Average Scaled	17	43	51	111	0.09	-0.71	0.48	-0.04	0.24	0.13	0.12	0.10
Zscore: Allen's Cognitive Level	17	43	51	111	-0.29	-0.19	0.17	-0.04	0.27	0.15	0.14	0.10

Structure Matrix		
	Function	
	1	2
Zscore: LSP_TOTAL_recode	.489*	0.025
Zscore: HoNOS Total score (re-coded)	.405*	0.164
Zscore: BPRS Total score	.401*	0.293
Zscore: SFS - Average Scaled	-.381*	-0.170
Zscore: SANS Total	.353*	0.158
Zscore: AUDIT Total score	-0.257	.911*
Zscore: Allen's Cognitive Level	-0.064	-.130*
Pooled within-groups correlations between discriminating variables and		
*. Largest absolute correlation between each variable and any discriminant		

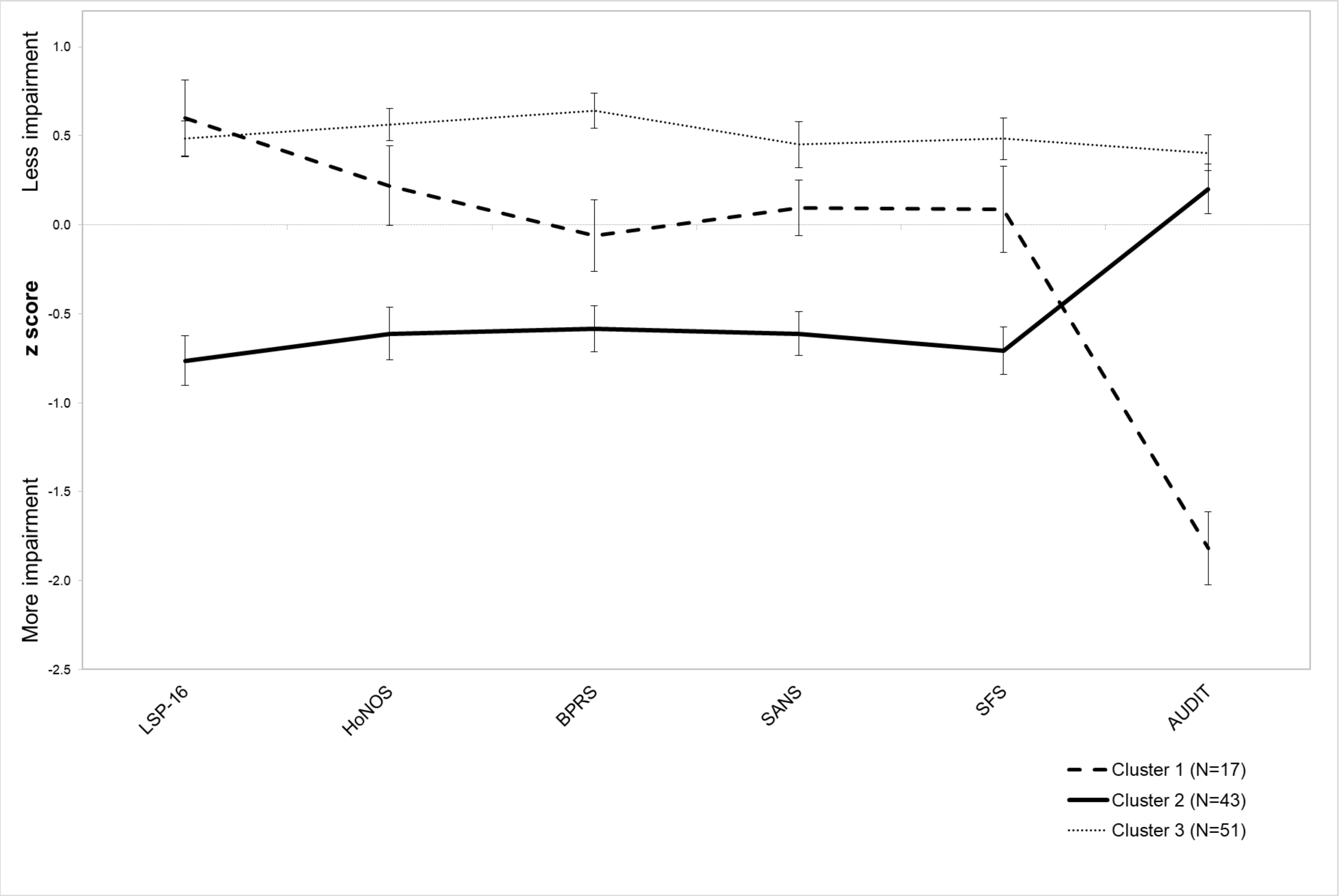

Less impairment
z score
More impairment
1.0
0.5
0.0
-0.5
-1.0
-1.5
-2.0
-2.5
LSP-16
HoNOS
BPRS
SANS
SFS
AUDIT
Cluster 1 (N=17)
Cluster 2 (N=43)
Cluster 3 (N=51)
